# Supplementary material for: Reduced choice-confidence in negative numerals
Source: PLoS One. 2022 Oct 3;17(10):e0272796. doi: 10.1371/journal.pone.0272796 (PMC9529092; doi:10.1371/journal.pone.0272796)

**Supplemental Information**

**Supplemental Figure 1** DDM fit Exp. 1. Response time distributions incorrect trials. Solid lines humans, dashed DDM. Humans made few mistakes, and their response times overlap, suggesting that incorrect trials were explained by other cognitive phenomena not present in the DDM model (e.g. attention lapses).

**
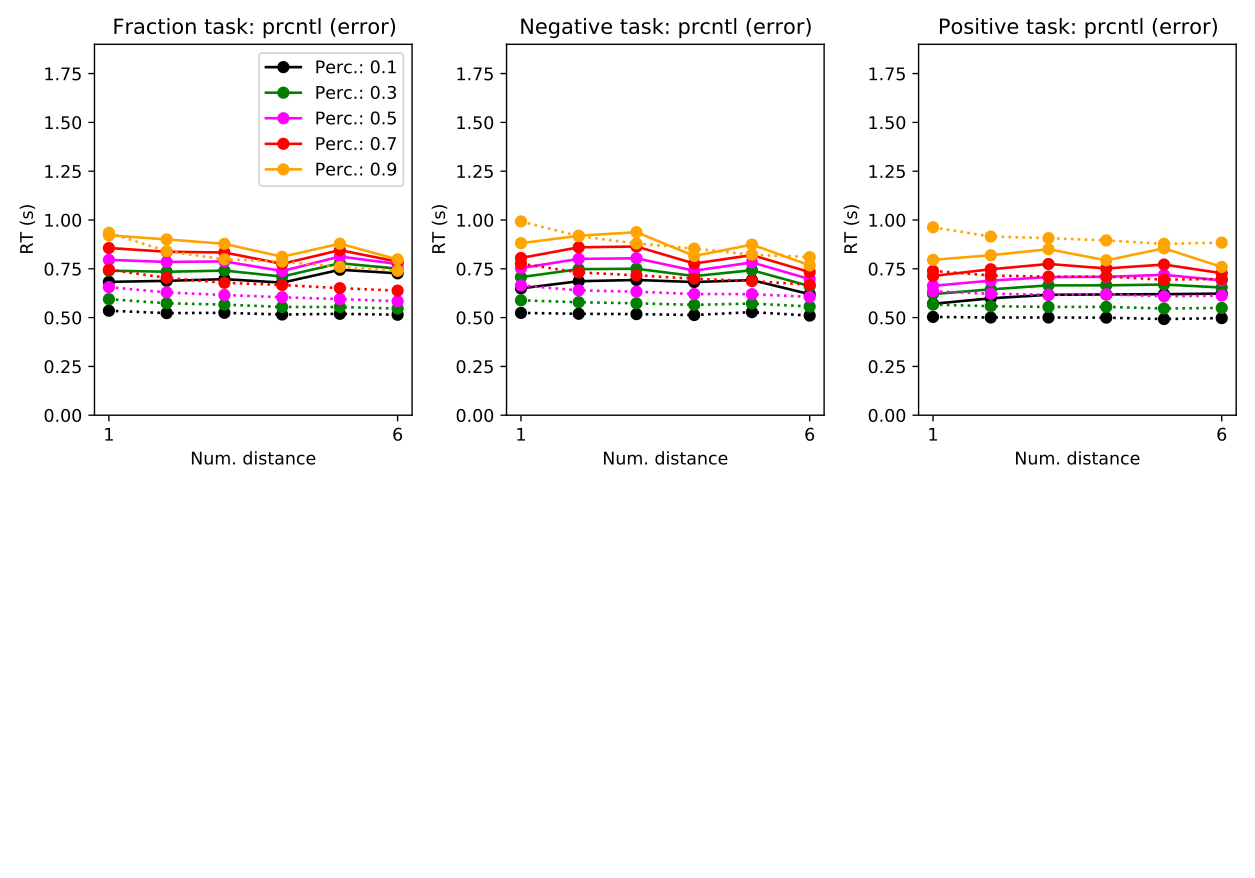
**

**Supplemental Figure 2.1** DDM fit Exp. 2. Response time distributions incorrect trials. Solid lines humans, dashed DDM.


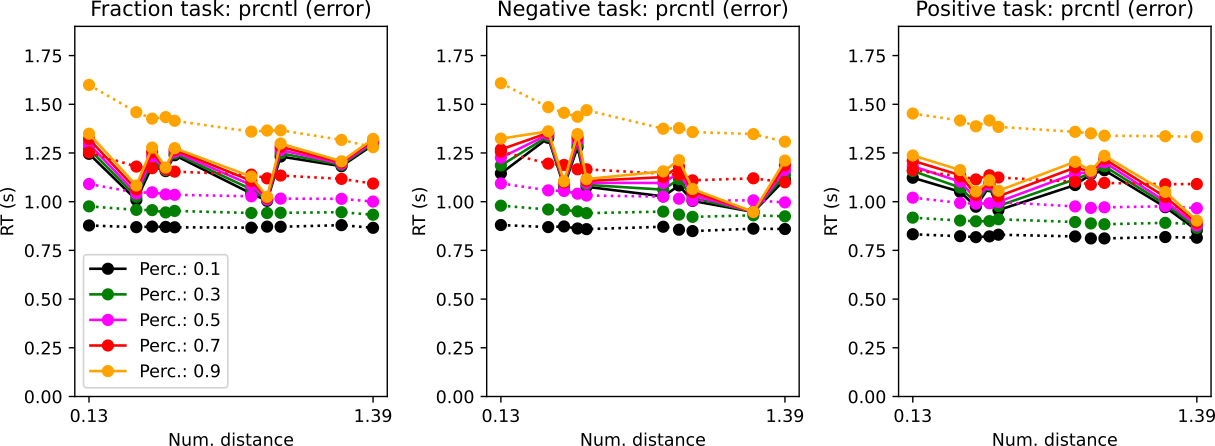


**Supplemental Figure 2.2** DDM fit Exp. 3. Response time distributions incorrect trials. Solid lines humans, dashed DDM.

**
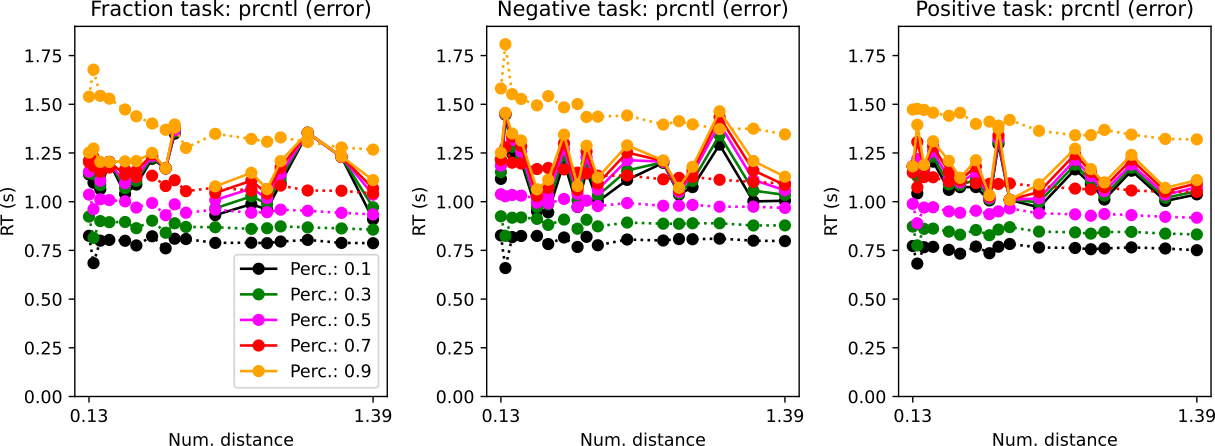
**

**Supplemental Figure 3-12. Random set of 10 individual fits. Each row is a RT percentile. Each column is a numerical distance. The model appropriately simulates responses times for individual participants.**

**
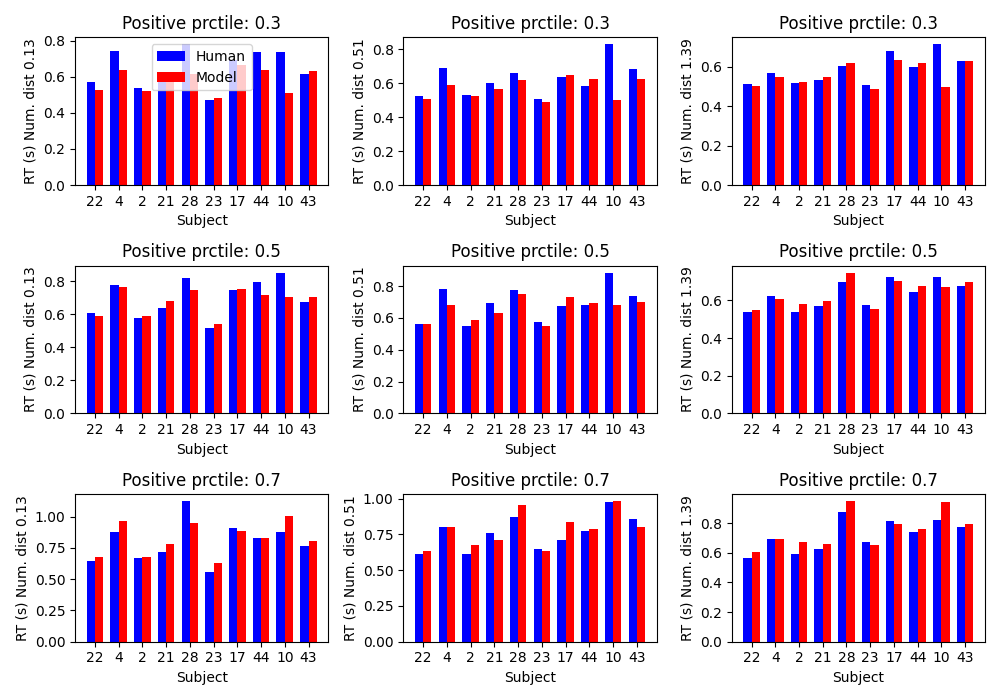
**

Supplemental Figure 3 (Exp. 1). Positive numerals

**
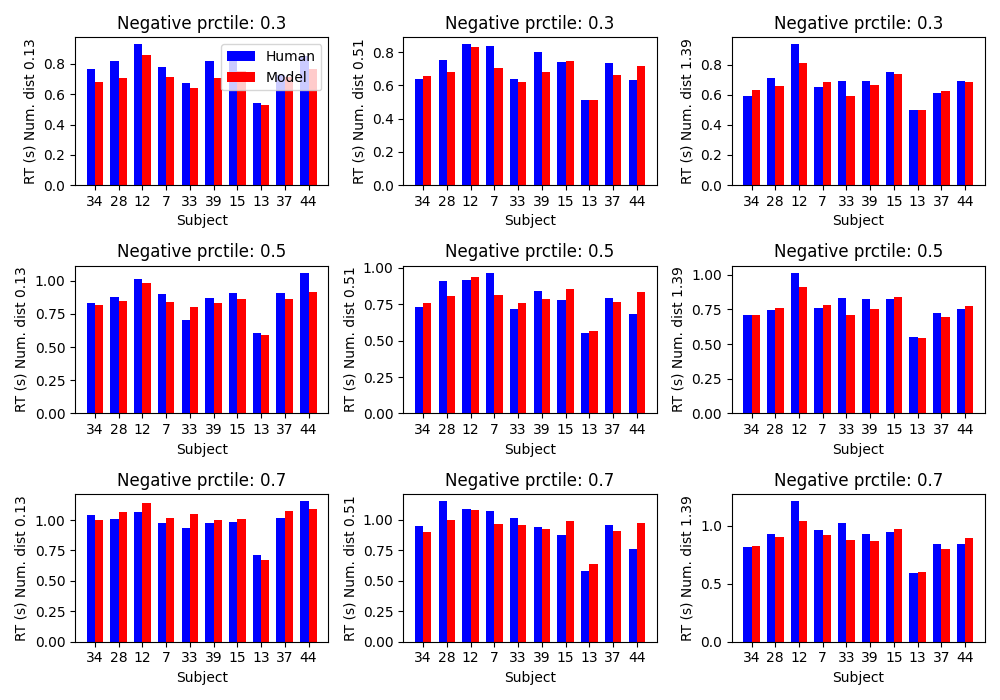
**

Supplemental Figure 4 (Exp. 1). Negative numerals

**
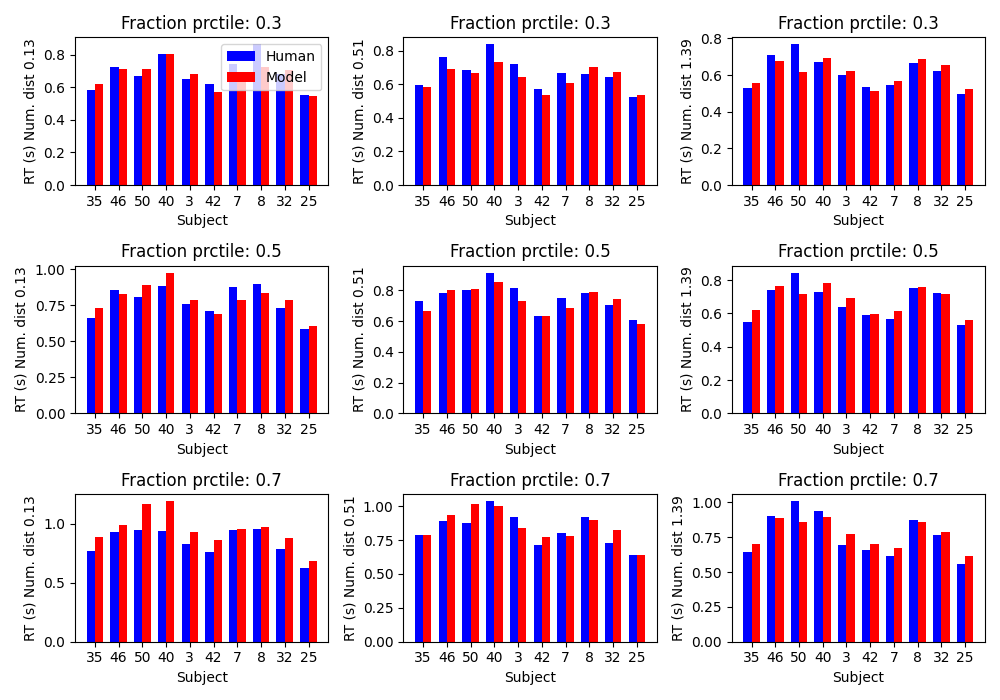
**

Supplemental Figure 5 (Exp. 1). 1/n fractions


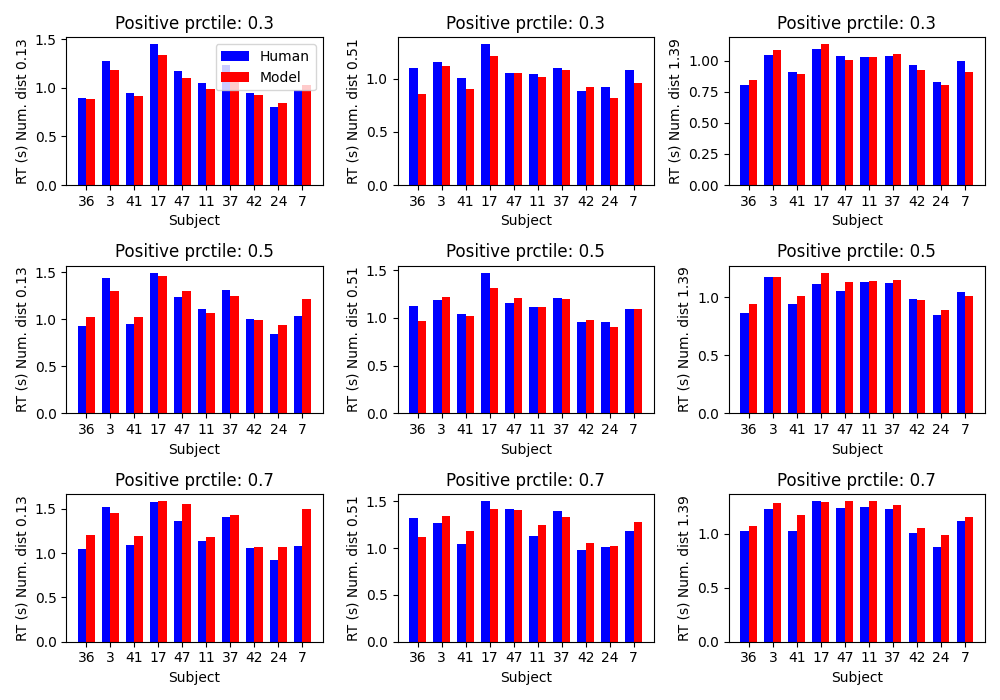


Supplemental Figure 6 (Exp. 2). Positive numerals


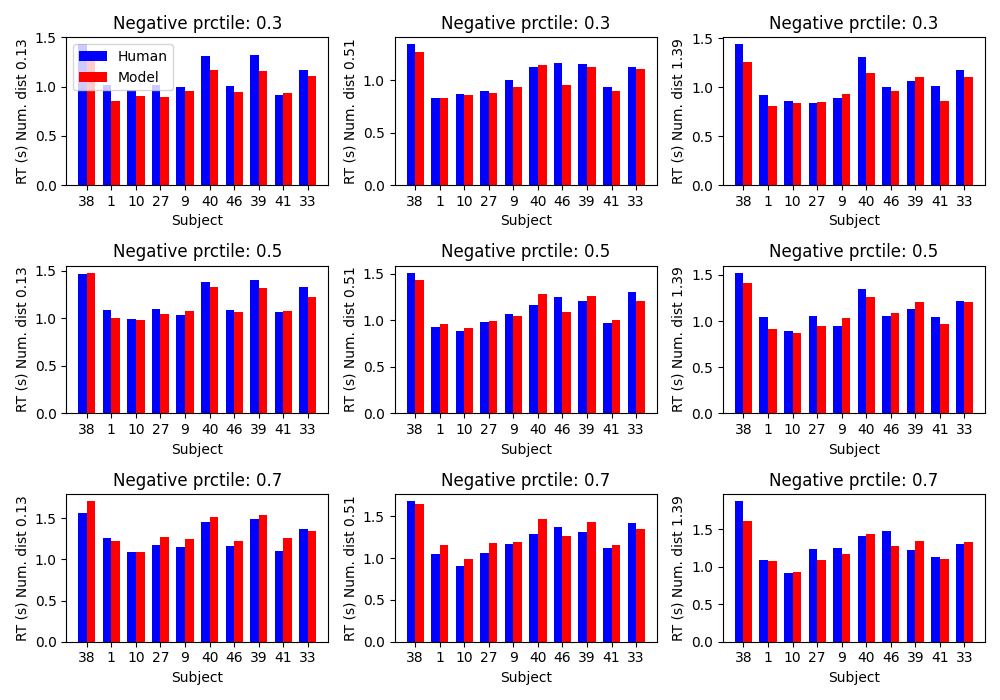


Supplemental Figure 7 (Exp. 2). Negative numerals


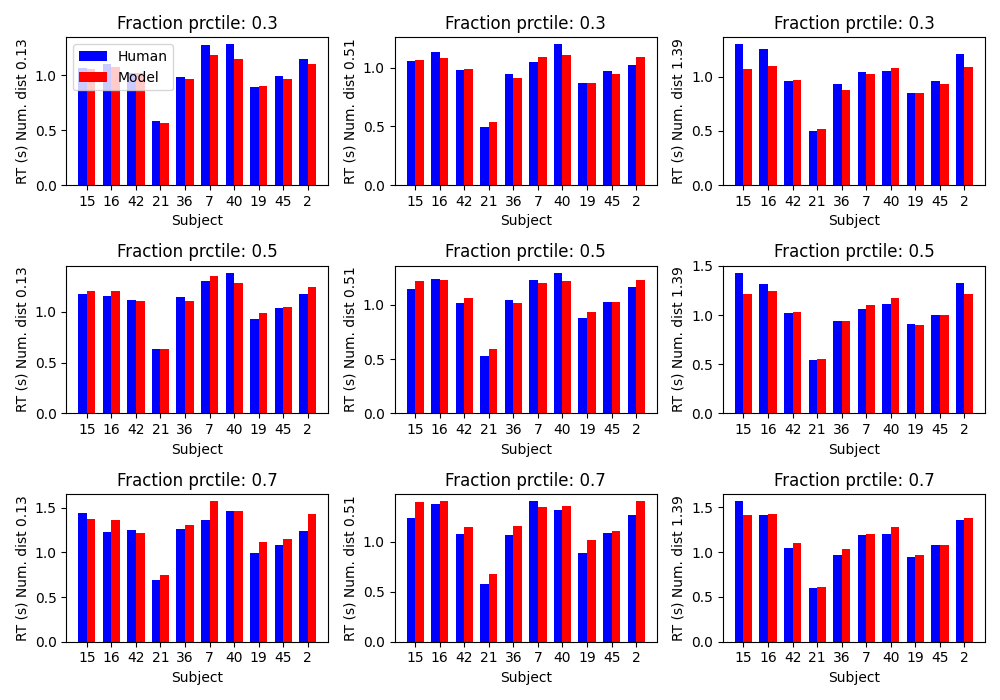
Supplemental Figure 8 (Exp. 2). 1/n fractions


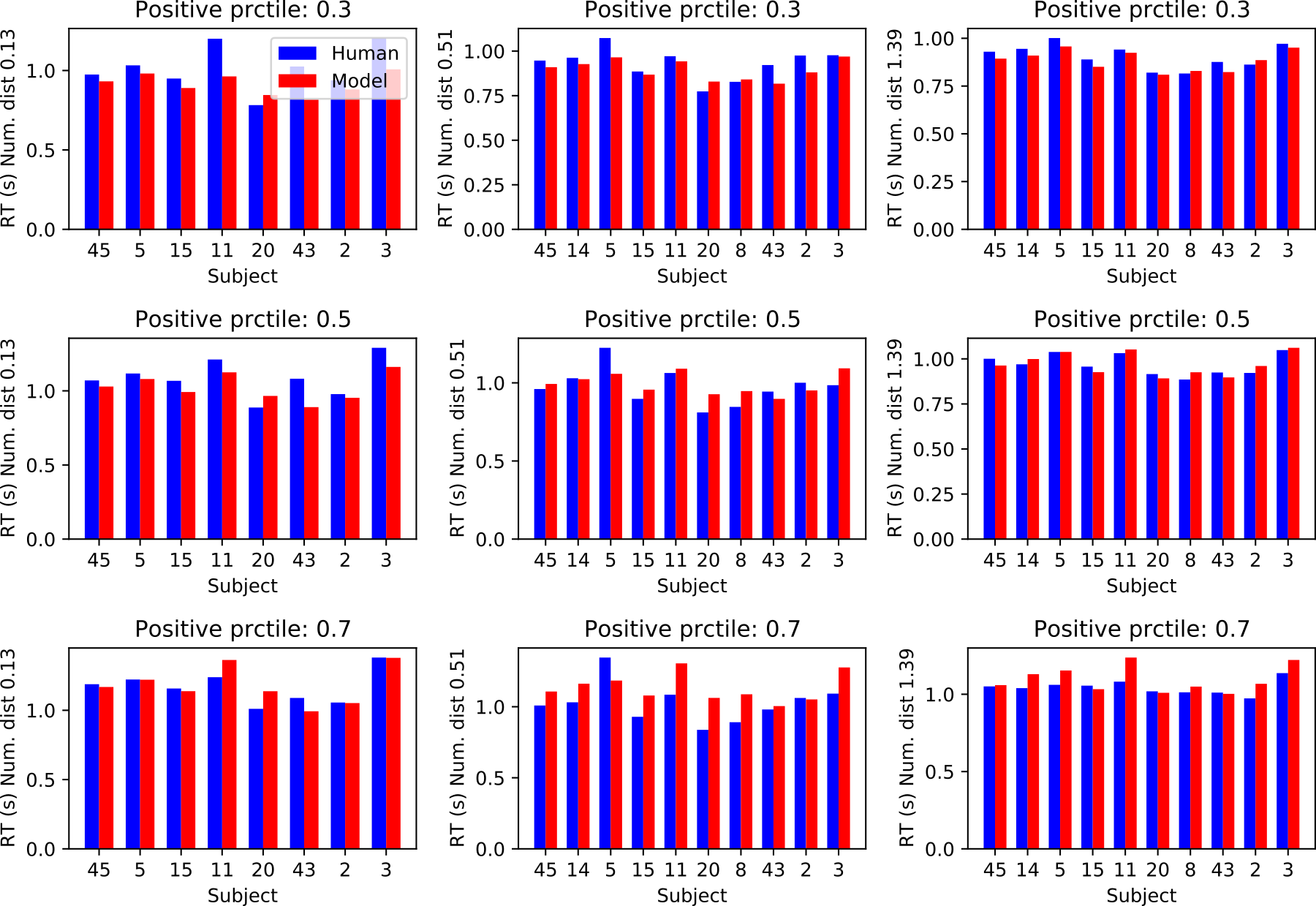


Supplemental Figure 9 (Exp. 3). Positive numerals


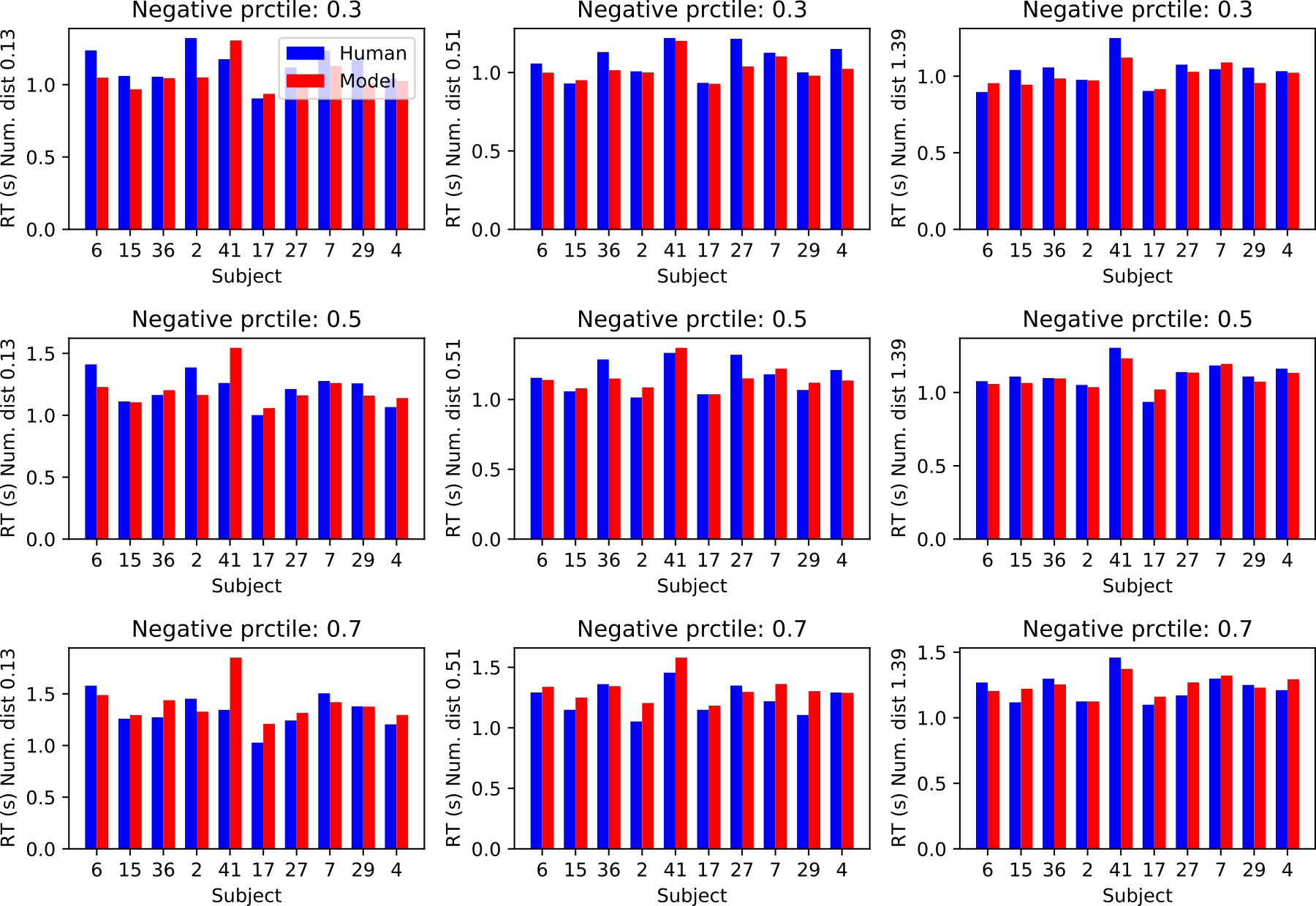


Supplemental Figure 10 (Exp. 3). Negative numerals


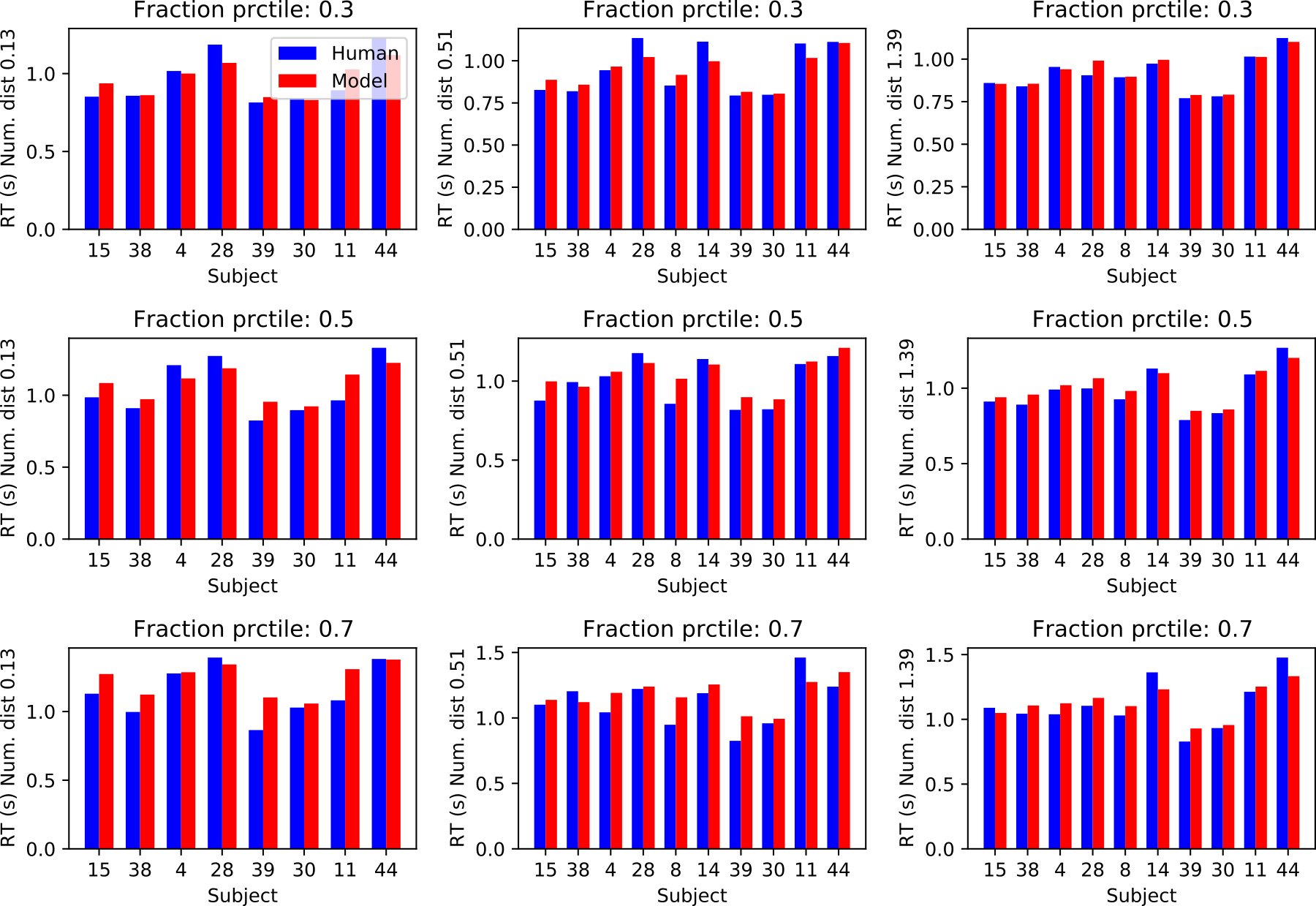


Supplemental Figure 11 (Exp. 3). 1/n fractions

**Supplemental Figure 12 - 14.** Change in decision dynamics. Each row has a parameter and each column a comparison between conditions. Stars are individual participants. From left to right: fraction minus negative, fraction minus positive, and negative minus positive trials. Each panel has two squares at the top. The left one is testing if the absolute difference between parameters is different from zero (red dashed line; one-sample t-test; orange if significant). The right one tests whether there was a dominant direction (paired sample t-test; green if significant). A white square indicates that the t-test was not significant. Significance of the t-tests is corrected for the 15 comparisons (Holm-Sidak).


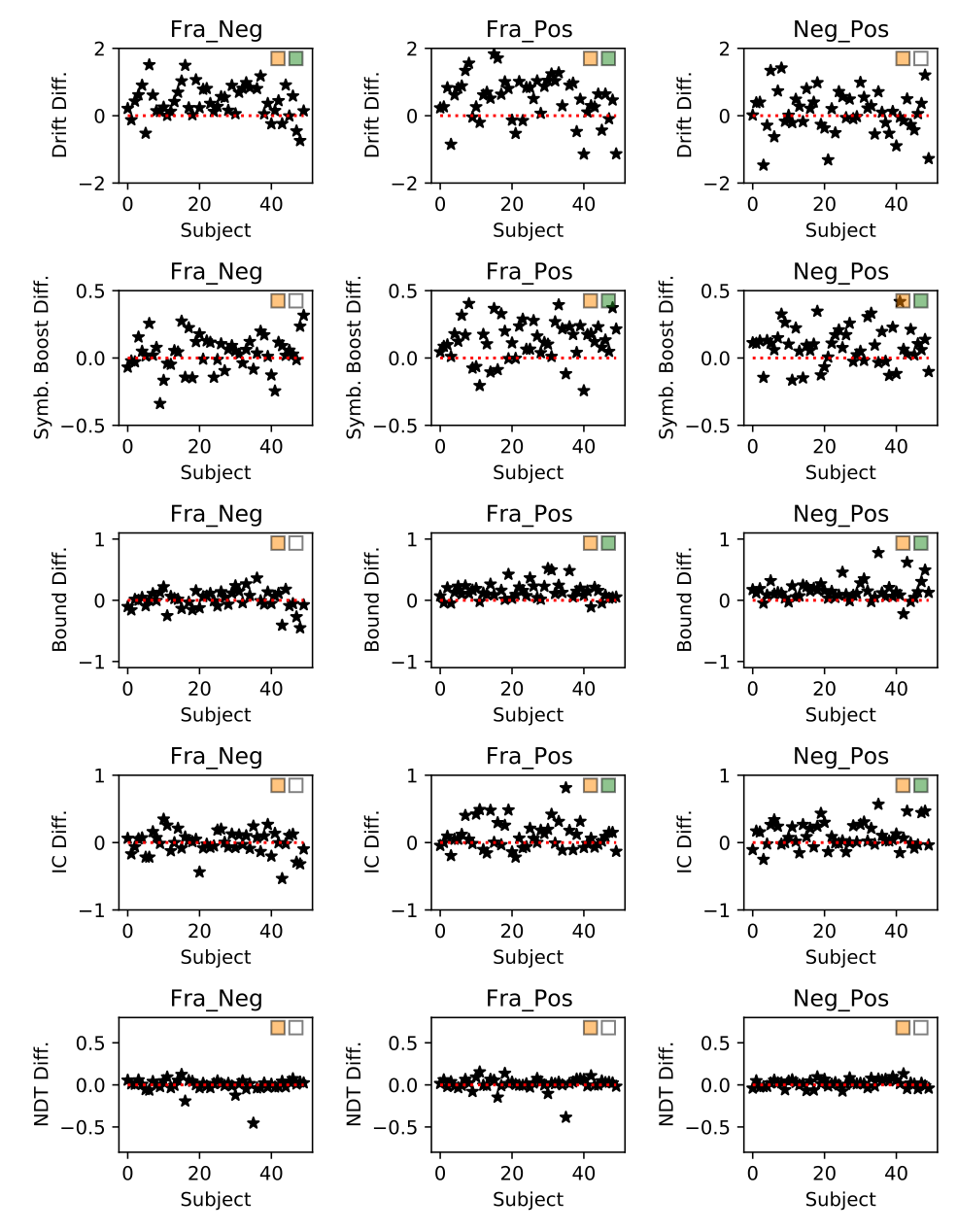


Supplemental Figure 12. Experiment 1


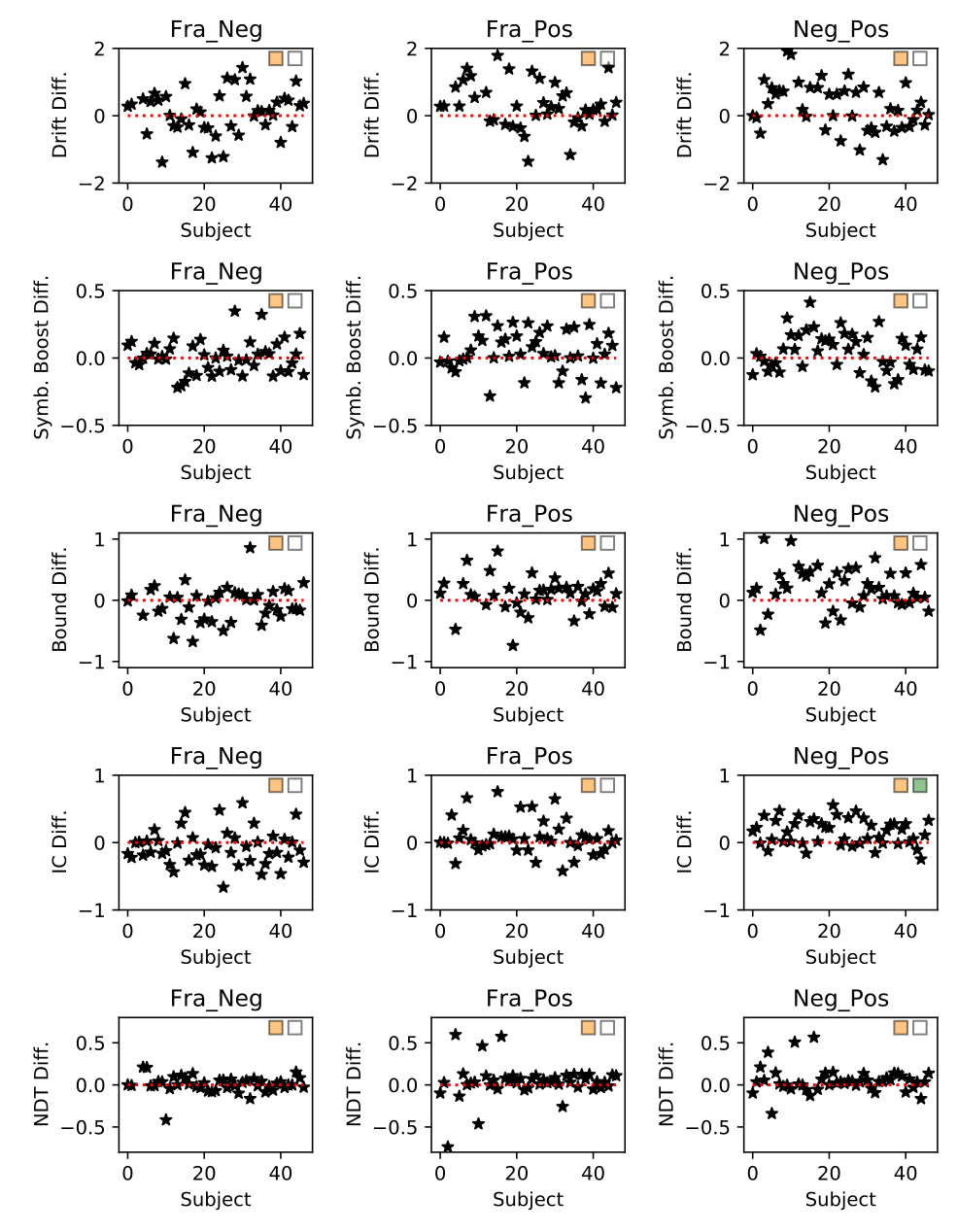


Supplemental Figure 13. Experiment 2


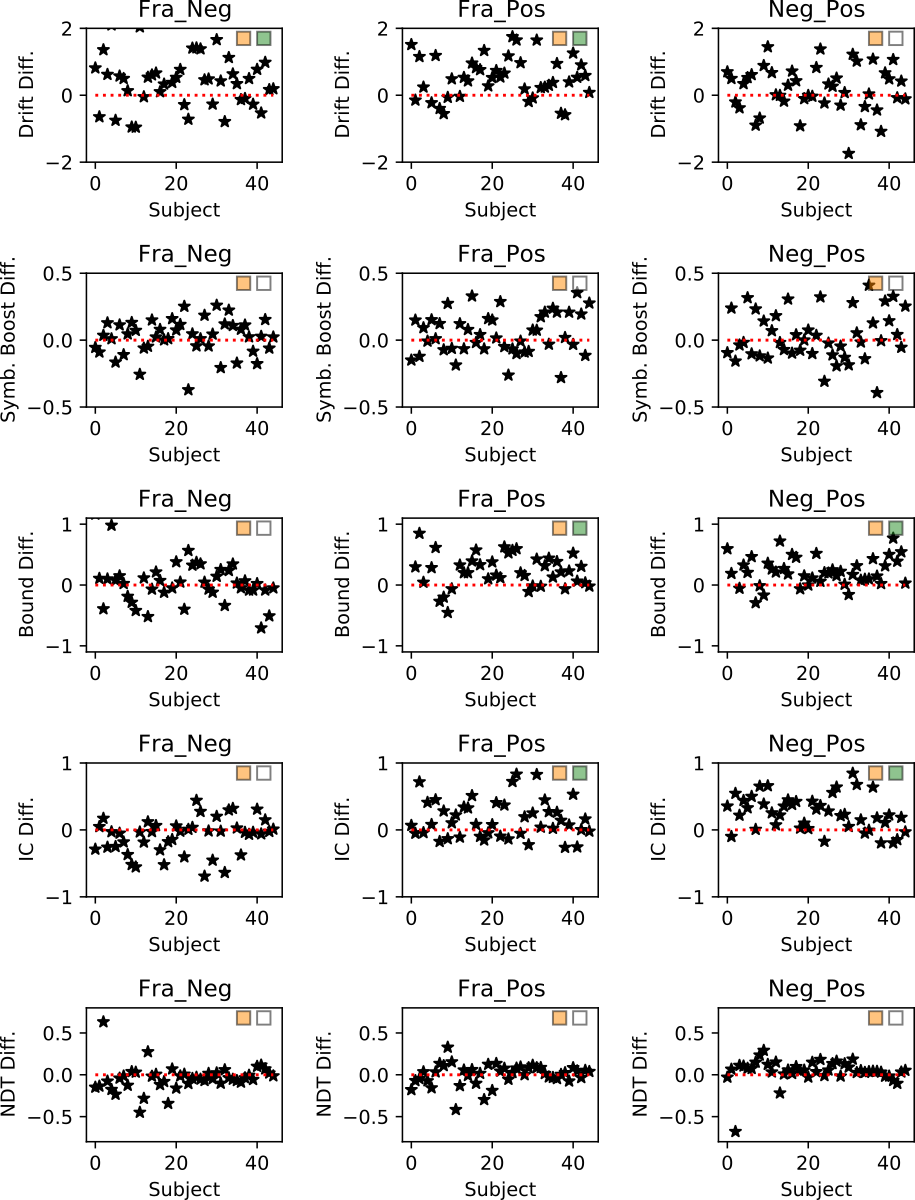


Supplemental Figure 14. Experiment 3

**Supplemental Table 1.** Regression full sample Exp. 3.

| Supp. Table 1. Exp 3. Fixed Effects Estimation Summary | | |  |  |  |  |
| --- | --- | --- | --- | --- | --- | --- |
| Dep. Variable: | Max Press (normed) | Log-likelihood | 3468.7 |  |  |  |
| Estimator: | FE | F-statistic (robust): | 47.048 |  |  |  |
| Cov. Estimator: | Robust | P-value | 0 |  |  |  |
| No. subj: | 49 | Distribution: | F(6,16353) |  |  |  |
| No. Observations: | 16408 | No. pars: | 7 |  |  |  |
| BIC: | -6869 | BIC vs Linear: | -3 |  |  |  |
|  | Par. | Std.Err | t | p | Low CI | High CI |
| Intercept | 0.47 | 0.02 | 25.87 | 0 | 0.44 | 0.51 |
| 1/n | -0.01 | 0 | -2.33 | 0.02 | -0.02 | 0 |
| Neg. | -0.01 | 0 | -2.87 | 0 | -0.02 | 0 |
| Correct | 0.08 | 0.02 | 5.05 | 0 | 0.05 | 0.12 |
| Num. dist | -0.01 | 0.02 | -0.68 | 0.5 | -0.05 | 0.03 |
| Dist:Correct | 0.02 | 0.02 | 1.08 | 0.28 | -0.02 | 0.06 |
| RT | 0.09 | 0.01 | 12.46 | 0 | 0.08 | 0.1 |

Including the four subjects with low performance (<85% correct) increases the sample to 49. The overall results are similar to the regression with the reduced sample of 45.

**Supplemental Figure 15.** Example of the pulse-like form of the Arduino sensor output. Notice that each trial looks like a peak. This means that participants had a brief contact with the sensor. Also, that there is variability in subject’s pressure. (here we plot the raw Arduino signal, before any standardization). Top: Exp. 2 participant; Bottom: Exp. 3 participant. We tried to improve sampling rate in Exp. 3 by dropping some markers sent by the Arduino, and the peaks tend to look a bit smoother. All analyzed trials and participants in Supplemental Figure 18 and 19.


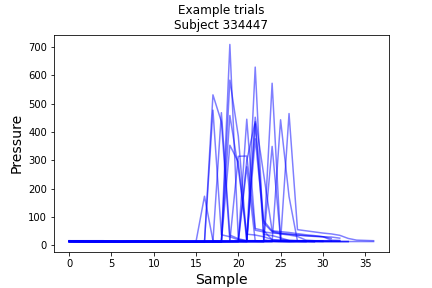


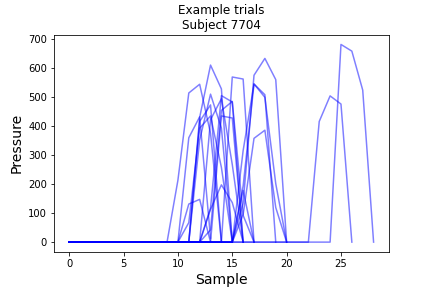


**Supplemental Figure 16.** Arduino diagram and board code.

**
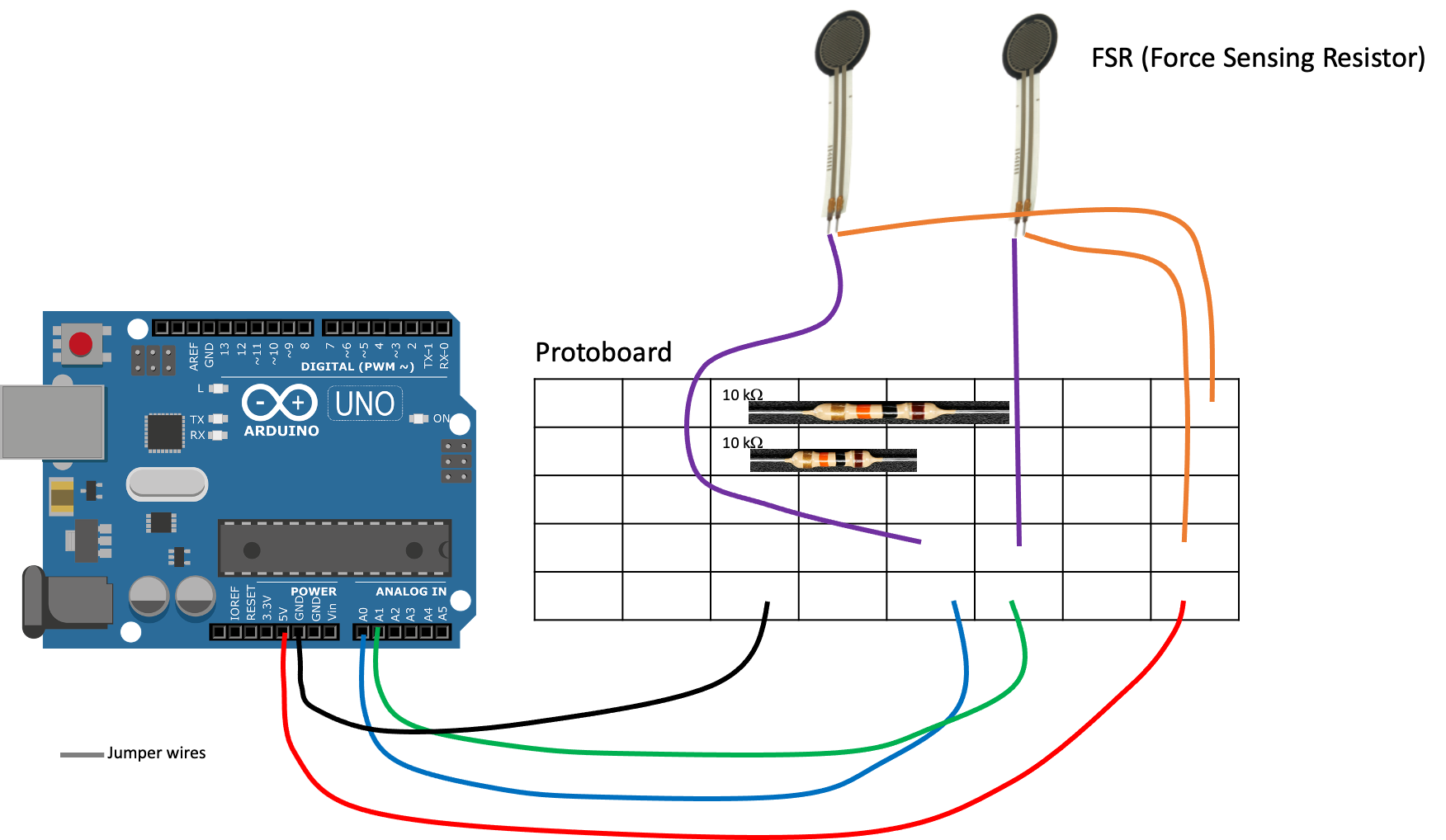
**

int fsrAnalogPin1 = 0;

int fsrAnalogPin2 = 1;

float analogReading;

void setup() {

Serial.begin(9600);

pinMode(fsrAnalogPin1, OUTPUT);

pinMode(fsrAnalogPin2, OUTPUT);

}

void loop() {

Serial.println("plh1");

Serial.println(999999, DEC);

Serial.println("fsr1");

analogReading = analogRead(fsrAnalogPin1);

Serial.println(analogReading, DEC);

Serial.println("plh2");

Serial.println(999999, DEC);

Serial.println("fsr2");

analogReading = analogRead(fsrAnalogPin2);

Serial.println(analogReading, DEC);

}

**Supplemental Figure 17.** DDM confidence properties experiments 1 and 3.

**
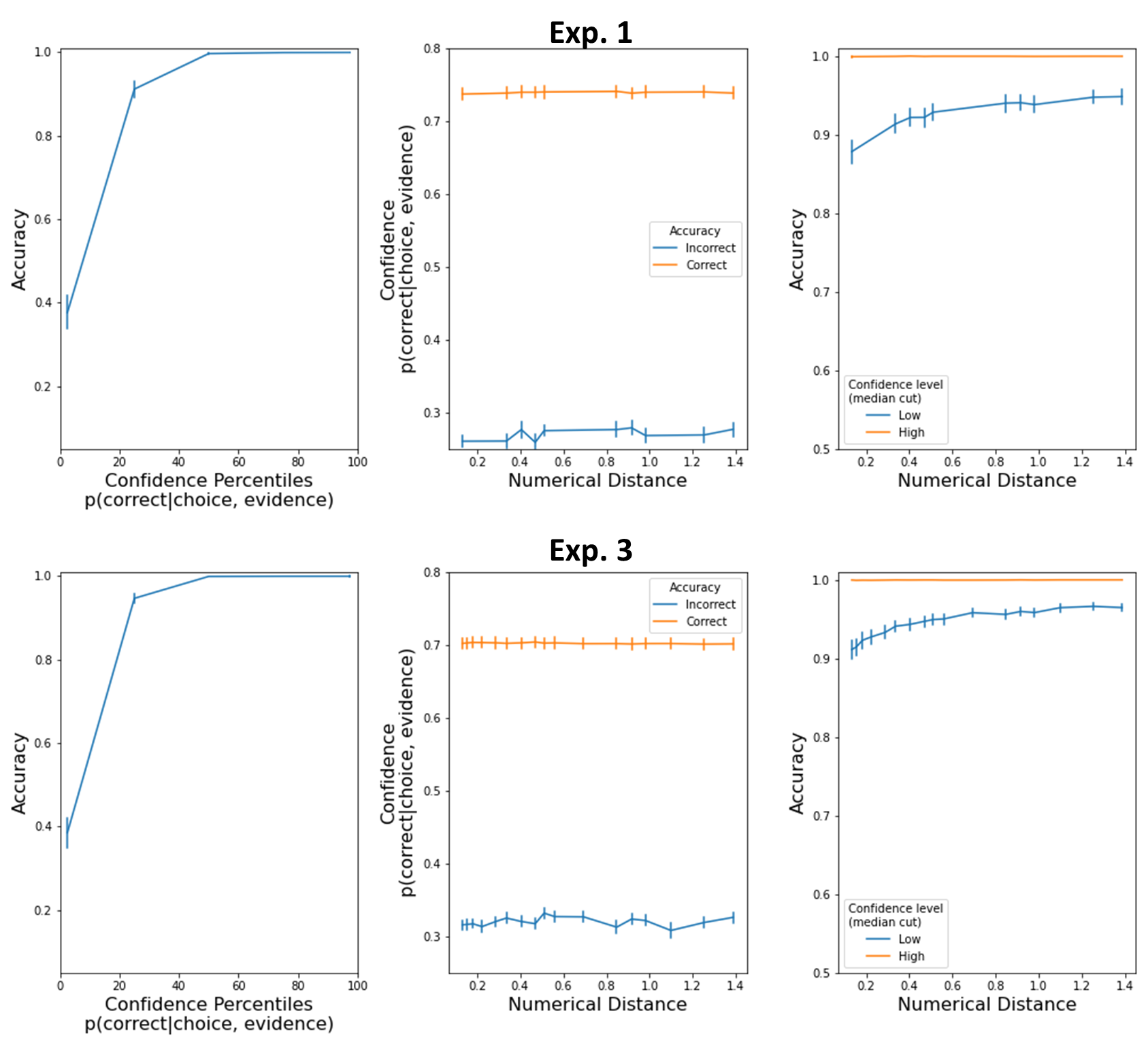
**

**Supplemental Figure 18.** All included trials by subject (Exp. 2). We excluded participant 33443 (responded all trials with the keyboard, not with the Arduino).


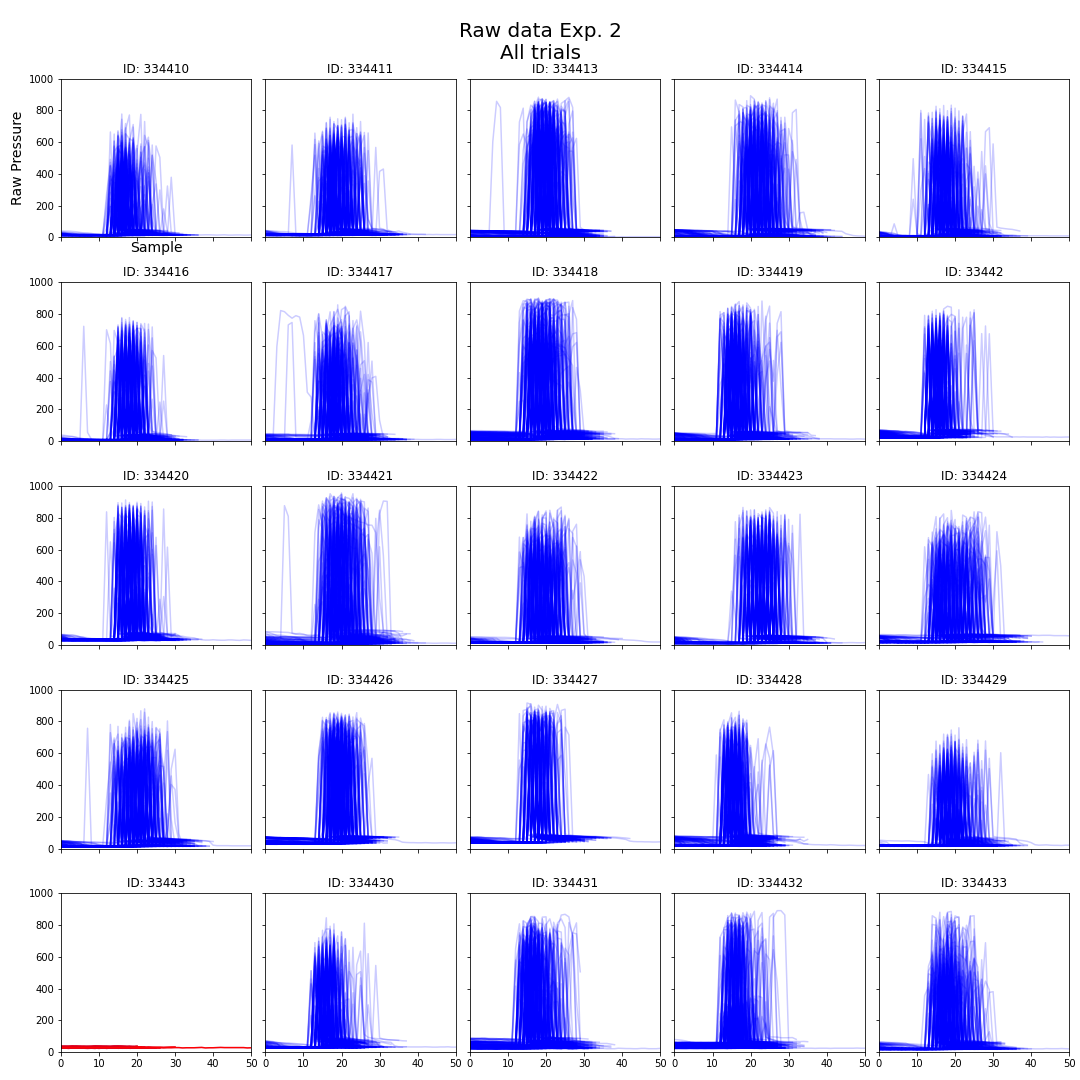


**
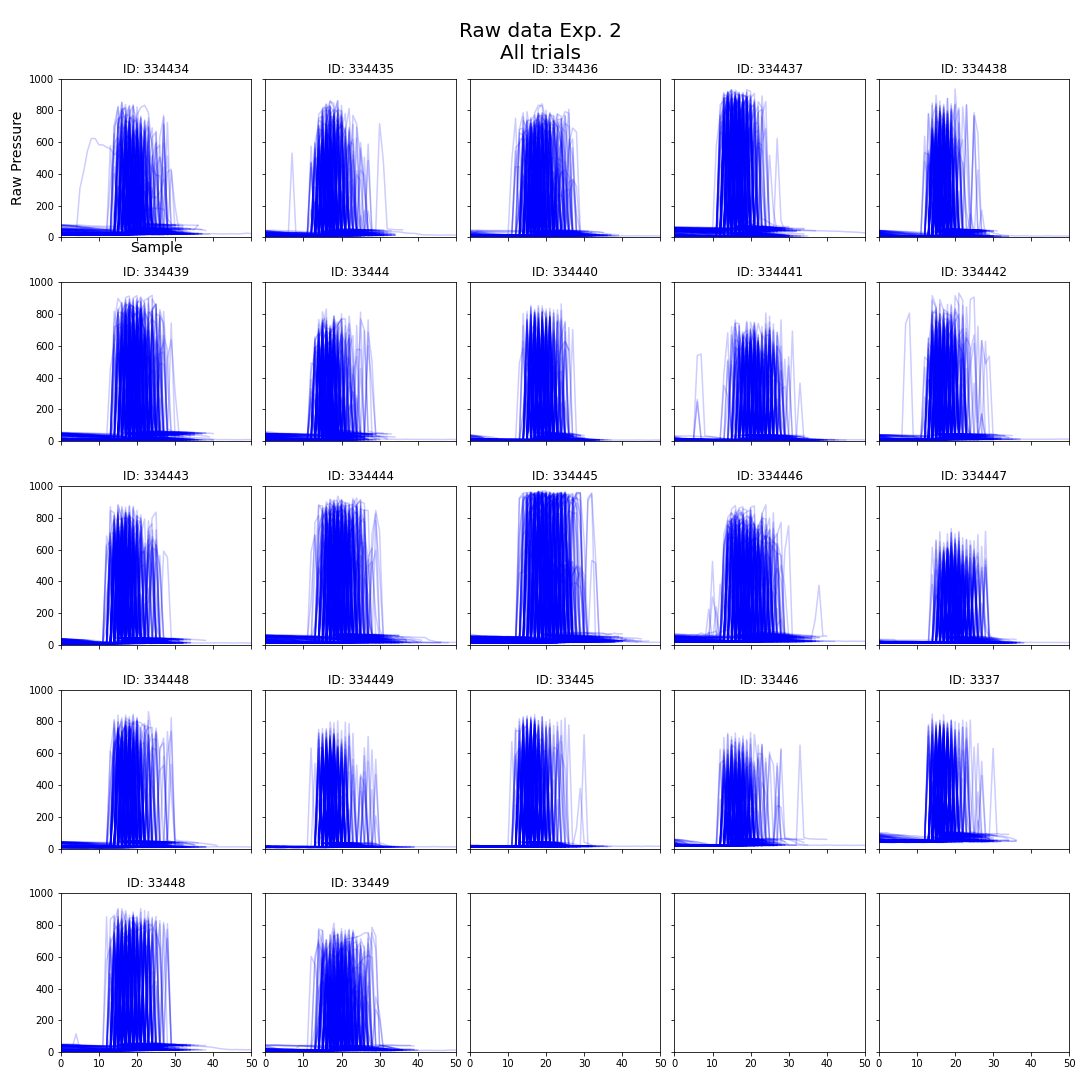
**

**Supplemental Figure 19.** All included trials by subject (Exp. 3).


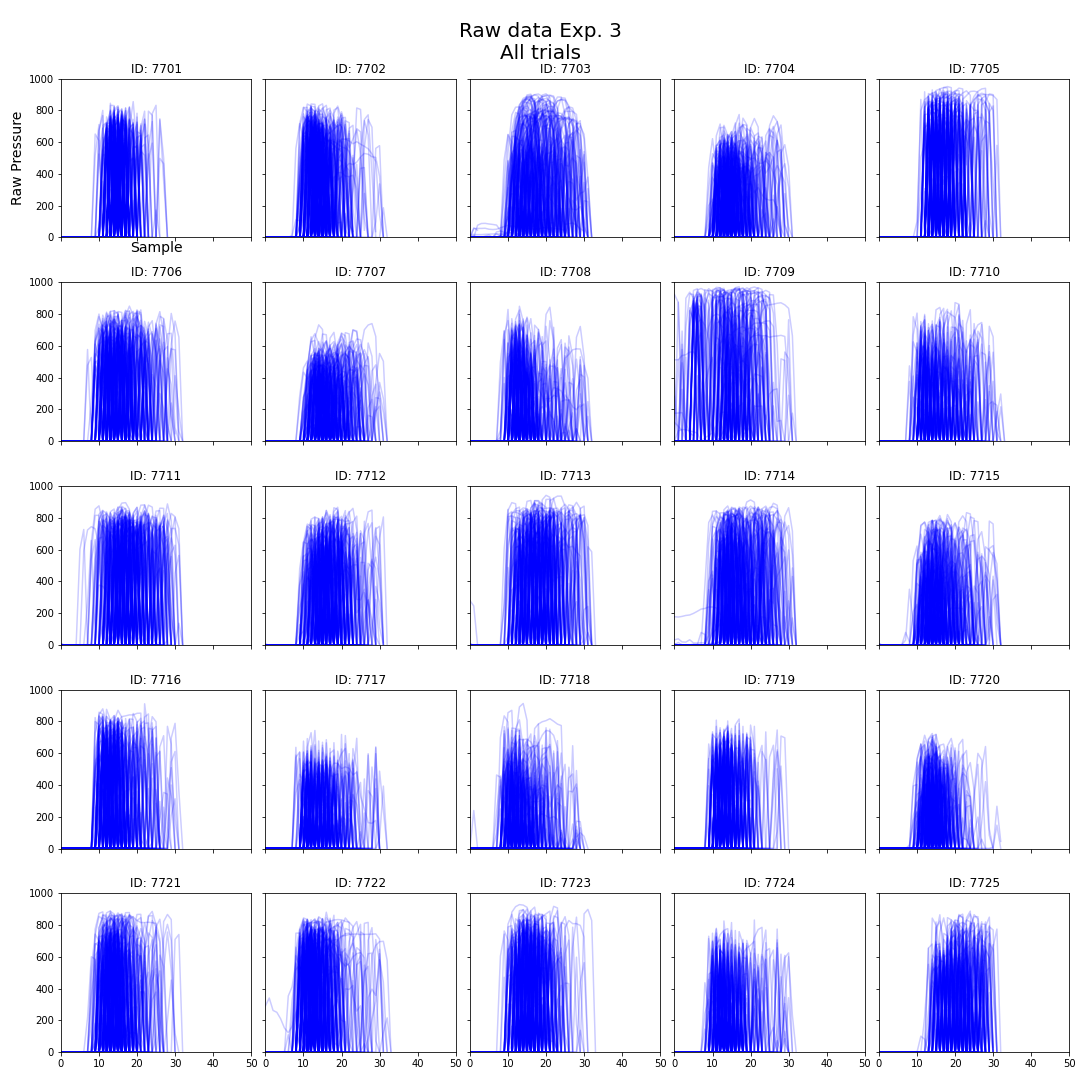


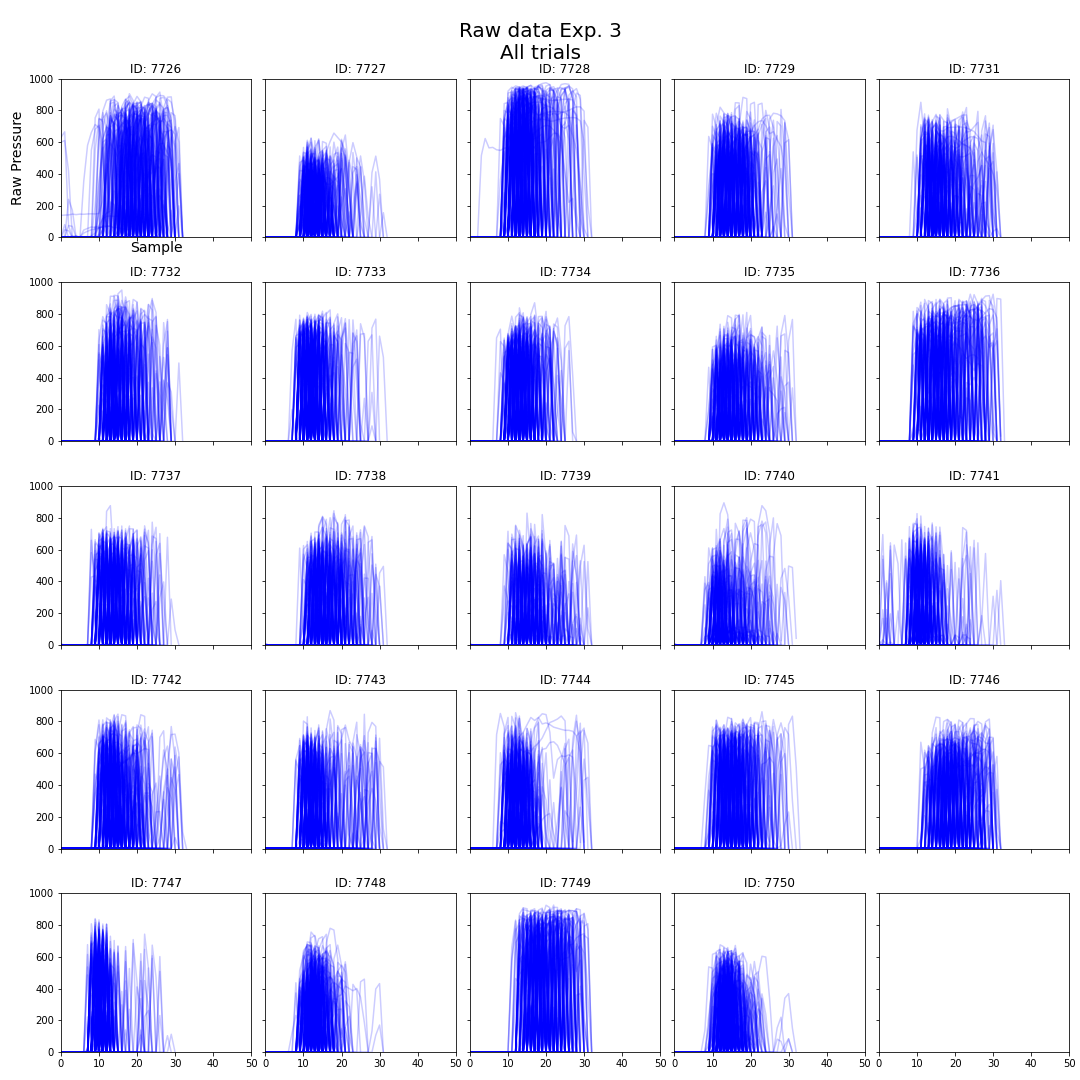

Supplement: S1 File — (DOCX) [file pone.0272796.s001.docx]
