# Supplementary material for: Reduced choice-confidence in negative numerals
Source: PLoS One. 2022 Oct 3;17(10):e0272796. doi: 10.1371/journal.pone.0272796 (PMC9529092; doi:10.1371/journal.pone.0272796)
Supplement: Attachment — Submitted filename: Reviewers_1_PONE.docx [file pone.0272796.s002.docx]

PONE-D-21-25521

Reduced choice-confidence in negative numerals

PLOS ONE

Dear Dr. Alonso-Diaz,

Thank you for submitting your manuscript to PLOS ONE. After careful consideration, we feel that it has merit but does not fully meet PLOS ONE’s publication criteria as it currently stands. Therefore, we invite you to submit a revised version of the manuscript that addresses the points raised during the review process.

I received two reviews about your manuscript. Especially reviewer #1 raised some serious methodological concerns that need to be addressed. I encourage you to amend the manuscript according to the suggestions, taking special care of the points raised by reviewer #1. Note that this can imply additional experimental work, according to how the comment about a potential design flaw is addressed.

When sublitting your revison, please double check figures and upload high quality artwork, current figures are barely readable. 

Please submit your revised manuscript by Mar 17 2022 11:59PM. If you will need more time than this to complete your revisions, please reply to this message or contact the journal office at [plosone@plos.org](mailto:plosone@plos.org). When you're ready to submit your revision, log on to https://www.editorialmanager.com/pone/ and select the 'Submissions Needing Revision' folder to locate your manuscript file.

Please include the following items when submitting your revised manuscript:

- A rebuttal letter that responds to each point raised by the academic editor and reviewer(s). You should upload this letter as a separate file labeled 'Response to Reviewers'.
- A marked-up copy of your manuscript that highlights changes made to the original version. You should upload this as a separate file labeled 'Revised Manuscript with Track Changes'.
- An unmarked version of your revised paper without tracked changes. You should upload this as a separate file labeled 'Manuscript'.

If you would like to make changes to your financial disclosure, please include your updated statement in your cover letter. Guidelines for resubmitting your figure files are available below the reviewer comments at the end of this letter.

If applicable, we recommend that you deposit your laboratory protocols in protocols.io to enhance the reproducibility of your results. Protocols.io assigns your protocol its own identifier (DOI) so that it can be cited independently in the future. For instructions see: [https://journals.plos.org/plosone/s/submission-guidelines#loc-laboratory-protocols](https://nam10.safelinks.protection.outlook.com/?url=https%3A%2F%2Fjournals.plos.org%2Fplosone%2Fs%2Fsubmission-guidelines%23loc-laboratory-protocols&data=04%7C01%7Calonsosantiago%40javeriana.edu.co%7C69e446115e354eb63ddf08d9e4e230b9%7Cdaf7990e8a3f409c9b762a5475098000%7C0%7C0%7C637792482426711039%7CUnknown%7CTWFpbGZsb3d8eyJWIjoiMC4wLjAwMDAiLCJQIjoiV2luMzIiLCJBTiI6Ik1haWwiLCJXVCI6Mn0%3D%7C3000&sdata=fFZp8MjHY5E2qcNWEGV0WG6j7uDXRp4Qnyi%2FjyTC1kE%3D&reserved=0). Additionally, PLOS ONE offers an option for publishing peer-reviewed Lab Protocol articles, which describe protocols hosted on protocols.io. Read more information on sharing protocols at [https://plos.org/protocols?utm_medium=editorial-email&utm_source=authorletters&utm_campaign=protocols](https://nam10.safelinks.protection.outlook.com/?url=https%3A%2F%2Fplos.org%2Fprotocols%3Futm_medium%3Deditorial-email%26utm_source%3Dauthorletters%26utm_campaign%3Dprotocols&data=04%7C01%7Calonsosantiago%40javeriana.edu.co%7C69e446115e354eb63ddf08d9e4e230b9%7Cdaf7990e8a3f409c9b762a5475098000%7C0%7C0%7C637792482426711039%7CUnknown%7CTWFpbGZsb3d8eyJWIjoiMC4wLjAwMDAiLCJQIjoiV2luMzIiLCJBTiI6Ik1haWwiLCJXVCI6Mn0%3D%7C3000&sdata=SjL%2FGTo4X%2BVo4KkRH1%2FgLdrM8oikOxa5z5zwfW2YXI0%3D&reserved=0).

We look forward to receiving your revised manuscript.

Kind regards,

Federico Giove, PhD
Academic Editor
PLOS ONE

Journal requirements:

When submitting your revision, we need you to address these additional requirements.

1. Please ensure that your manuscript meets PLOS ONE's style requirements, including those for file naming. The PLOS ONE style templates can be found at

https://journals.plos.org/plosone/s/file?id=wjVg/PLOSOne_formatting_sample_main_body.pdf and

[https://journals.plos.org/plosone/s/file?id=ba62/PLOSOne_formatting_sample_title_authors_affiliations.pdf](https://nam10.safelinks.protection.outlook.com/?url=https%3A%2F%2Fjournals.plos.org%2Fplosone%2Fs%2Ffile%3Fid%3Dba62%2FPLOSOne_formatting_sample_title_authors_affiliations.pdf&data=04%7C01%7Calonsosantiago%40javeriana.edu.co%7C69e446115e354eb63ddf08d9e4e230b9%7Cdaf7990e8a3f409c9b762a5475098000%7C0%7C0%7C637792482426711039%7CUnknown%7CTWFpbGZsb3d8eyJWIjoiMC4wLjAwMDAiLCJQIjoiV2luMzIiLCJBTiI6Ik1haWwiLCJXVCI6Mn0%3D%7C3000&sdata=RfcciBg9gBVzr3CCtH%2B22vs6%2FPDXSi0D9YE3o7P%2FNWM%3D&reserved=0)”

2. Please provide additional details regarding participant consent. In the ethics statement in the Methods and online submission information, please ensure that you have specified what type you obtained (for instance, written or verbal, and if verbal, how it was documented and witnessed). If your study included minors, state whether you obtained consent from parents or guardians. If the need for consent was waived by the ethics committee, please include this information.

3. Thank you for stating the following in the Funding Section of your manuscript:

“The study received funding from the university.”We note that you have provided funding information that is not currently declared in your Funding Statement. However, funding information should not appear in the Acknowledgments section or other areas of your manuscript. We will only publish funding information present in the Funding Statement section of the online submission form.

Please remove any funding-related text from the manuscript and let us know how you would like to update your Funding Statement. Currently, your Funding Statement reads as follows:

“S.A. received an early career grant from the university (Pontificia Universidad Javeriana. ID PPTA 8329; https://www.javeriana.edu.co/vicerrectoria-de-investigacion/). The funders had no role in study design, data collection and analysis, decision to publish, or preparation of the manuscript”

Please include your amended statements within your cover letter; we will change the online submission form on your behalf.

4. Your ethics statement should only appear in the Methods section of your manuscript. If your ethics statement is written in any section besides the Methods, please delete it from any other section.

5. Please include captions for your Supporting Information files at the end of your manuscript, and update any in-text citations to match accordingly. Please see our Supporting Information guidelines for more information: http://journals.plos.org/plosone/s/supporting-information.

[Note: HTML markup is below. Please do not edit.]

Reviewers' comments:

Reviewer's Responses to Questions

**Comments to the Author**

1. Is the manuscript technically sound, and do the data support the conclusions?

The manuscript must describe a technically sound piece of scientific research with data that supports the conclusions. Experiments must have been conducted rigorously, with appropriate controls, replication, and sample sizes. The conclusions must be drawn appropriately based on the data presented.

Reviewer #1: No

Reviewer #2: Yes

2. Has the statistical analysis been performed appropriately and rigorously?

Reviewer #1: No

Reviewer #2: Yes

3. Have the authors made all data underlying the findings in their manuscript fully available?

The [PLOS Data policy](https://nam10.safelinks.protection.outlook.com/?url=http%3A%2F%2Fwww.plosone.org%2Fstatic%2Fpolicies.action%23sharing&data=04%7C01%7Calonsosantiago%40javeriana.edu.co%7C69e446115e354eb63ddf08d9e4e230b9%7Cdaf7990e8a3f409c9b762a5475098000%7C0%7C0%7C637792482426711039%7CUnknown%7CTWFpbGZsb3d8eyJWIjoiMC4wLjAwMDAiLCJQIjoiV2luMzIiLCJBTiI6Ik1haWwiLCJXVCI6Mn0%3D%7C3000&sdata=PaJouxlLH%2BB0oawdEd%2BpkwYCv%2BmnOzYc6%2BrJmb4Fv04%3D&reserved=0) requires authors to make all data underlying the findings described in their manuscript fully available without restriction, with rare exception (please refer to the Data Availability Statement in the manuscript PDF file). The data should be provided as part of the manuscript or its supporting information, or deposited to a public repository. For example, in addition to summary statistics, the data points behind means, medians and variance measures should be available. If there are restrictions on publicly sharing data—e.g. participant privacy or use of data from a third party—those must be specified.

Reviewer #1: No

Reviewer #2: Yes

4. Is the manuscript presented in an intelligible fashion and written in standard English?

PLOS ONE does not copyedit accepted manuscripts, so the language in submitted articles must be clear, correct, and unambiguous. Any typographical or grammatical errors should be corrected at revision, so please note any specific errors here.

Reviewer #1: No

Reviewer #2: Yes

5. Review Comments to the Author

Please use the space provided to explain your answers to the questions above. You may also include additional comments for the author, including concerns about dual publication, research ethics, or publication ethics. (Please upload your review as an attachment if it exceeds 20,000 characters)

Reviewer #1: Summary

**We want to start by thanking you for your detailed review, is something we truly appreciate, and hope to fully address your main concerns below and in the new manuscript.**

The authors report statistical modeling of two data sets in which adults performed magnitude comparisons with positive, negative, and fraction numerals. Their main point is that the second data set revealed weaker maximum response force for negative compared to the other number types.

Evaluation
While the main message is newsworthy, the ms fails to integrate this finding adequately into the current literature and consequently lacks important methodological and analytical detail. Furthermore, a potential flaw in the design might account for the result and needs to be addressed. A substantial revision is necessary before this ms could perhaps make a useful contribution to the field.

Major Problems
1) LITERATURE: The authors seem unaware of the distinction between kinematics and kinetics of movement. While they cite a range of kinematic studies (mouse tracking, pointing), they completely overlooked the cognitive literature on force production, beginning with Abrams & Balota (1991) and extending to the recent modeling work of Miklashevsky et al. (2021) in the numerical domain. This omission results in rather superficial reporting for the “novel” dependent measure, both in terms of data collection and data analysis (see below). Another example is the authors’ referring to Dotan’s work for log compression (p. 21) but the same authors have since revoked this account (Dotan & Dehaene, 2016).

- **Indeed, we had a bias towards kinematic studies, which we are more familiar with; this is our first force paper. We are grateful for these new references relating force and number cognition. In the introduction we tried to summarize the overall findings of this literature. page 4**
- **We also drop any mention that our paper uses a “novel” dependent measure. We are now aware that it is not the case.**
- **Regarding the Dotan paper of 2013, we no longer use it in the new manuscript.**

2) METHODS AND DESIGN: The information contained on p. 6-8 is incomplete and needs to be massively expanded and systematized (separate sections for participants, apparatus, stimuli, design, procedure) in order to allow proper appreciation and replication. A key point to elaborate is the recording and subsequent analysis of force data (see Miklashevsky et al., 2021 and references therein for the complexity of this topic) to help readers understand the choice and extraction of the specific force measure used.

- **We extend the explanation of the apparatus and measure (page 7) and provide a diagram of the Arduino setup in supplemental information (Supp. Fig. 13).**

The relationship between number magnitude and force should also be reported to relate this work to the current debate.

- **Tables 3 report this relationship. It was not significant. We did find lower button pressure for negative numerals than for positive numerals, suggesting that the nature of the number representations do affect motor planning. We discuss this in page 27.**

I list here several other specific omissions:
a) There is contradictory information about the range of numbers used (either 2-15 or 2,3,5,7,8; and why not 4 and 6?) and the specific items and their frequency that resulted in the reported number of trials.

- **We put a table with the actual numbers and frequencies (page 9).**
- **We improved the explanation on why the set 2,3,5,7, and 8, and the explanation on why we distinguished between dummy (2-15) and non-dummy trials (2,3,5,7,and, 8) (pages 7-9)**
- **We ran a new experiment with the full set 2,3,4,5,6,7,8.**

b) What is a “dummy trial”?

- **A trial that exposes participants to a wider range of numbers but, and this is important, we do not include in data analysis. We clarify in the new manuscript why these trials are important to avoid anchor strategies (which we also explain better in the new manuscript and see our response to c) below). Pages 7-9**

c) What is meant by “anchor strategies” (illustrative example and references needed)?

- **In the set of numbers 1 to 9, 1 and 9 are the extreme anchors. Thus, if participants experience that set of numbers, every time they see a 1 or 9 in the pair they don’t have to compute any distance, they just pick the other number (if 1) or 9.**
- **Thus, dummy trials include 9 (the range of dummy trials was 2 to 15) but we do not analyze them (also because in dummy trials there are two-digit numbers).**
- **Including dummy trials was a conservative approach to avoid strategic behavior so that participants mostly based their judgments on numerical distance.**

d) The sample size is not justified, either a priori or retrospectively.

- **We now further clarify in the methods a retrospective reason (we preregistered 50), and give a rationale why we do not calculate post-hoc power. Page 6**
- **Sample size for the new experiment was capped at 50.**

e) There seems to be no specific ethics approval for this study (as indicated by a reference number), merely a general statement that authors complied with ethical regulations.

- **Sorry if this was not clear. We now further emphasize that the study was approved by an actual committee and the reference number. Page 7**

f) The data collection was embedded into a series of related tasks, apparently intended to prime “inversion” (motion perception, categorization) that is not sufficiently well reported to permit understanding of possible spill-over effects; ideally, absence of spill-over should be formally reported in terms of non-significant order effects.

- **We change all the regressions to random effects panel regressions to include the between-subjects factor of order (page 9 and 10). Fixed-effects regressions by definition do not accept between-subject variables. The overall findings that numeral type affected button pressure holds, even after controlling for order. Moreover, the presence of theoretically relevant effects of confidence in button pressure do not disappear after controlling for order (Tables 2,3,4,5)**
- **We ran a new experiment where participants only did the numeral comparison task and the results were similar.**

3) RESULTS
a) While there is extensive statistical modeling, some basics remain opaque because of lack of descriptives, such as reporting of average RT or accuracy in the text. One example is the differential distance effect in accuracy (p. 11) and speed;

- **We now include average and standard deviations for accuracy (page 14) and response time (page 18), by numeral type.**
- **Also, Figure 4 has the average accuracy and response times by percentiles, both for participants and the DDM model.**

also, the authors confused “two samples” with “two-sided” testing (p. 10, bottom).

- **We did mean two-sample t-test (i.e. a paired independent-samples t test). We clarify this in the new manuscript (Table 5, pages 20 and 21).**

b) The Figures in the ms are of poor quality, making even the identification of axes labels impossible. This is unprofessional. All I was able to notice is that the authors erroneously used the unit “percent” for a probability scale.

- **We are sorry for this. We submitted high quality figures however it seems that the ones rendered by the submission system on the manuscript were low quality ones.**
- **The high-quality ones had to be accessed via the link in the upper left corner of the page containing each figure.**
- **In case the rendering system fails, we now uploaded the manuscript with figures in place to psyarxiv: https://psyarxiv.com/sdfb9**

4) DISCUSSION:
a) A potential flaw in the design might account for the result and needs to be addressed. Specifically, the decision to feed back errors through color changes (p.7) established specific color transition probabilities that would be effective at the start of each new trial. This may well be the very mechanisms by which “incorrect trials priors somehow moved” (p. 18) that the authors speculate about. Should this not be ruled out with a control experiment?

- **We now include in the regressions a dummy variable controlling if the current trial was preceded by an incorrect response. The overall results were the same, which discards a simple color transition effect (Tables 2,3,4).**
- **Moreover, there are really few incorrect trials (approx. 5%), so such transitions were experienced rarely in most participants.**
- **Finally, we also ran a new experiment without feedback or any color changes and we obtained similar results**

b) The authors never fulfill their promise (from p. 10) to discuss implications of the assumed seriality of processes (I found line 6 on p. 21 as the only return to this important issue)

- **We agree that this is an important issue but we do not have the means to confirm the seriality. We apologize if in the original manuscript there was any indication that our paper was about seriality. For this revision, we drop any promise and discussion, and just state in the introduction that it is an important assumption of the drift diffusion framework. We think is important to make this assumption transparent for future researchers using the DDM model.**

Minor Issues: These largely relate to the writing style; in light of their sheer number it is worth highlighting them:
- The authors frequently state incomplete comparisons. Already the third sentence needs to be completed “…worse for negative numerals than for …”; there are numerous such instances throughout the ms.

- **Hopefully we completed all comparisons (we apologize if we still have any in the new manuscript)**

- The ms contains a large number of grammatically problematic formulations. A pertinent example that misleads readers is on page 8, line 4 from the bottom, where the authors stated ”It includes...” but should have stated “They include…” because it is NOT the decision variable that includes encoding and responding-related times.

- **We fixed this and (hopefully) similar ones.**

- The writing is often opaque because of colloquial style (e.g., first new paragraph on p. 19). But already the very first sentence “Negatives are essential in mathematics” requires elaboration to rule out photographic negatives.

- **Sorry for this. We did not try to be opaque and agree that science should be clear. We had colleagues read the original manuscript but it was not enough. For this new submission we tried to revise any opaque writing. Also, even though we know is just but an example you brought, we changed the expression “negatives” with the more specific “negative numerals” or “negative numbers”.**

- There are some illogical statements. For example, the sentences 5 and 6 of the Intro are contradictory because lack of variability prevents strong correlations.

- **We erased those sentences. We wanted to say that high accuracy relates to high confidence, and this is a usual finding. However, we decided to drop that idea as it is not critical for the argument.**

On page 9 various cognitive processes are ascribed to the minus B parameter.

- **We are not sure if we understand your point here. B is compression and minus is the rotation to the negative portion. We now further clarify this in the new manuscript.**

- Further intransparency results from the use of different labels for the same concept (1/positive numerals, inverted numbers, inverted problems, later 1/n fractions) and lack of definition of acronyms such as BIC (p.9).

- **We changed all 1/n (or similar) to 1/positive numerals.**
- **We now define the acronym BIC.**

- The ms should be checked for typos (e.g., p. 9: “striping” should be “stripping”, p. 18: “between response times” should be “with response times”)

- **Done (hopefully).**

References
Abrams, R. A., & Balota, D. A. (1991). Mental chronometry: Beyond reaction time. Psychological Science, 2, 153-157.
Dotan, D., & Dehaene, S. (2016). On the origins of logarithmic number-to-position mapping. Psychological Review, 123(6), 637–666. doi:10.1037/rev0000038
Miklashevsky, A., Lindemann, O. & Fischer, M.H. (2021). The Force of Numbers: Investigating Manual Signatures of Embodied Number Processing. Front. Hum. Neurosci. 14:590508. doi: 10.3389/fnhum.2020.590508

Reviewer #2: This paper presents experiments and models on the confidence of decision (post-decision estimate of correct) in the case of negative numbers.
It is an important issue, as many evidences both from human children and animals suggest that their coding is different from small positive numerosities and numbers.
I find particularly interesting that authors complement the study with human participants and the model.
About this, I suggest to add a wider reference on models proposed in numerical cognition. More in general, as it is a very wide field, adding more references can be useful.
Results confirm authors' hypothesis and are interesting for a wide audience; I suggest to stress the contribution of the model in the discussion.

- **Thanks for your comments and noting the significance of the paper. We appreciate it.**
- **We now stress the contribution of the model. In particular, in regard to confidence Pages 27-28.**
- **We do not compare our model to other non-ddm models (e.g. Huber, et al, 2016 connectionist model) because it will be too hard and speculative.**

6. PLOS authors have the option to publish the peer review history of their article ([what does this mean?](https://nam10.safelinks.protection.outlook.com/?url=https%3A%2F%2Fjournals.plos.org%2Fplosone%2Fs%2Feditorial-and-peer-review-process%23loc-peer-review-history&data=04%7C01%7Calonsosantiago%40javeriana.edu.co%7C69e446115e354eb63ddf08d9e4e230b9%7Cdaf7990e8a3f409c9b762a5475098000%7C0%7C0%7C637792482426711039%7CUnknown%7CTWFpbGZsb3d8eyJWIjoiMC4wLjAwMDAiLCJQIjoiV2luMzIiLCJBTiI6Ik1haWwiLCJXVCI6Mn0%3D%7C3000&sdata=plMFqPJC2X%2B9h1%2Fd42gq1ZkWN1%2FuHxdDKcweKAeUMfg%3D&reserved=0)). If published, this will include your full peer review and any attached files.


If you choose “no”, your identity will remain anonymous but your review may still be made public.


**Do you want your identity to be public for this peer review?** For information about this choice, including consent withdrawal, please see our [Privacy Policy](https://nam10.safelinks.protection.outlook.com/?url=https%3A%2F%2Fwww.plos.org%2Fprivacy-policy&data=04%7C01%7Calonsosantiago%40javeriana.edu.co%7C69e446115e354eb63ddf08d9e4e230b9%7Cdaf7990e8a3f409c9b762a5475098000%7C0%7C0%7C637792482426711039%7CUnknown%7CTWFpbGZsb3d8eyJWIjoiMC4wLjAwMDAiLCJQIjoiV2luMzIiLCJBTiI6Ik1haWwiLCJXVCI6Mn0%3D%7C3000&sdata=veaQbeTxmempUX9fYl%2FtPsKmIujm0hy5sXXY3QZH7oc%3D&reserved=0).

Reviewer #1: No

Reviewer #2: No

[NOTE: If reviewer comments were submitted as an attachment file, they will be attached to this email and accessible via the submission site. Please log into your account, locate the manuscript record, and check for the action link "View Attachments". If this link does not appear, there are no attachment files.]

While revising your submission, please upload your figure files to the Preflight Analysis and Conversion Engine (PACE) digital diagnostic tool, [https://pacev2.apexcovantage.com/](https://nam10.safelinks.protection.outlook.com/?url=https%3A%2F%2Fpacev2.apexcovantage.com%2F&data=04%7C01%7Calonsosantiago%40javeriana.edu.co%7C69e446115e354eb63ddf08d9e4e230b9%7Cdaf7990e8a3f409c9b762a5475098000%7C0%7C0%7C637792482426711039%7CUnknown%7CTWFpbGZsb3d8eyJWIjoiMC4wLjAwMDAiLCJQIjoiV2luMzIiLCJBTiI6Ik1haWwiLCJXVCI6Mn0%3D%7C3000&sdata=ZUFXVGme6EVDlO0ZaBeERGxWYsAe7dIW8v8GfSvKi%2Bg%3D&reserved=0). PACE helps ensure that figures meet PLOS requirements. To use PACE, you must first register as a user. Registration is free. Then, login and navigate to the UPLOAD tab, where you will find detailed instructions on how to use the tool. If you encounter any issues or have any questions when using PACE, please email PLOS at [figures@plos.org](mailto:figures@plos.org). Please note that Supporting Information files do not need this step.
