# Supplementary material for: Reduced choice-confidence in negative numerals
Source: PLoS One. 2022 Oct 3;17(10):e0272796. doi: 10.1371/journal.pone.0272796 (PMC9529092; doi:10.1371/journal.pone.0272796)
Supplement: Attachment — Submitted filename: Reviewers_2_PONE.docx [file pone.0272796.s003.docx]

Reviewer #1:

I have read the cover letter and the revised ms plus the supplementary document and my impression is mixed.

1. The authors made several adjustments and additions that strengthen the ms, including explanations of their idiosyncratic terminology (“dummy trials” for filler trials) and references to relevant papers that were previously omitted. However, repeatedly directing readers to Ganor-Stern & Tzelgov (2008) for the component model is still suboptimal because the paper by Huber (their Ref 5) constitutes its more recent development.

**We now extend the component model explanation with the Huber et al neural network (Intro.: Pages 3-4).**

Similarly, the introduction of “button pressure” as the measure of interest (on page 4) is immediately followed by references to “motor metrics”, i.e. kinematic studies, even though the “button pressure” is an isometric assessment without any kinematics involved. In my idiosyncratic view these are suboptimal revisions.

**We wanted to show to a general audience that “motor metrics” are used in cognition. We understand that those references were about kinematics. Thus, we now drop those kinematics references to avoid any confusion.**

2. More importantly, the methods descriptions are still not detailed enough to permit replication. Although stimulus ranges are now clear and the recording apparatus for “button pressure” is now explained in more detail, the sensitivity of this device is still unclear. If the range is 0.25 to 0.90, does this mean that 65 levels of pressure were discriminable

**The range 0.25-0.90 is continuous. There are more than 65 discrete levels.**

**The actual range from the raw data is 0.025 to 1 (Exp 2) and 0.05 to 1 (Exp. 3). The 0.25 – 0.9 range was after taking averages per numerical distances. In the new manuscript (Data analysis: Page 10) we decided that it was clearer if we present the ranges from the raw data because the right end is 1 and the left range is close to zero.**

while the actual force produced (in Pascal or Newton) remains unknown?

**The sensor detects pressure as a change in resistance. The details of the resistors and Arduino wiring are in the manuscript and supplemental information. The Arduino transforms those changes of resistance into a discrete signal between 0 and 1023. All subjects used the same Arduino setup (including voltage and resistors). Thus, we do not report Pascals or Newtons (the transformation from change in resistance to Newtons or Pascals is not trivial). Still, the change in resistance indeed relates to force; resistance-based pressure is used in many current devices including mobile phones and many portable devices.**

Another open issue, of fundamental importance for the later discussion about early attentional vs later conceptual effects: What was the sampling frequency (i.e. the temporal resolution of pressure measurements)?

**We now report the sampling frequency of the Arduino board in the manuscript (Apparatus, page 7-8) (approx. 26 hz but see manuscript for details).**

**It is not fast enough to fully disentangle early attention and later concepts (holistic magnitudes).**

**In the discussion we accept that it could be attention, but it would require some ad-hoc accommodations so that all our tables and results are about attention and not confidence.**

**As for concepts, we do think that confidence results speak about concepts, namely the existence of negative holistic magnitudes (see discussion section). However, we are also aware that our paper only brings partial clarity to negative magnitude processing. Our paper is just about confidence.**

3. Moreover, despite my extensive queries on this point, I still find only a SINGLE sentence that describes the force data analysis, namely “Data analysis. We used panel linear regressions to analyze the accuracy (linear probability model), response times up to two standard deviations from the mean (i.e., 95% of the trials), and button pressure”.

**We apologize for our lack of clarity; it is not our intention. In the first revision we tried to address all your comments, but we clearly fail. We hope that our responses below clarify our data analysis.**

This leaves open fundamental questions such as the time during which force was recorded or integrated,

**We analyzed signals as soon as the numerals appeared on screen and until the subject selected an option and was no longer pressing the force sensor (i.e. force resistance < low_pressure_threshold) (this for all trials). (Data Analysis: Page 10).**

the data filtering or trimming for this inherently noisy signal,

**We did not filter Arduino signals. When the sensor is pressed it sends a reading to Matlab based on the participant’s pressure. In the figure below, each trial has a stereotypical look: a peak. This means that participants had a brief contact with the sensor** **(here we plot the raw Arduino signal, before any standardization) (see all raw data in Supplemental Figures 18 and 19).**

**We filtered data on the behavior side. We reported in the manuscript two criteria: response time greater than 2 standard deviations from the mean of all the data and accuracy less than 85%.**

**We did detect an anomaly thanks to your comment. We plotted all the raw pressure data and we detected one subject who used the keyboard instead of the Arduino sensors. The pressures of subj. 33443 (Exp. 2) were always at baseline levels (Supp. Figure 18). This subj did respond accurately to the task, meaning that they used the keyboard (we explain this on Page 6 of the new manuscript).**

**The reason this could happen was that we coded the first experiment (Exp. 1) so that subjects had to use the keyboard. In Exp. 2 we used the same base code (plus the Arduino stuff) and even though we verbally explained to all subjects to use the Arduino sensors, we kept, by mistake, the original instructions of Exp. 1 on screen. Subj. 33443 was the only subject (across Exp. 2 and 3) who mistakenly used the keyboard (see Supplemental Figure 18). We excluded this subject from this revision. Importantly, the main pattern of results did not change.**

or the computation of parameters for analysis, such as average or peak force per trial, or many other candidates. None of this is explained.

**For this revision we now provide a concrete example for further clarity (Data Analysis: Page 10). Specifically, we used for analysis peak force during the trial, normalized to the max peak force during the whole session as a normalizing constant (separately for the left and right sensor). For instance, if subject X max peak force to the left during his whole session was 984, then all left pressures during the session were divided by 984. We used this metric in the tables. That is, the regression parameters were obtained with standardized peak pressure. We now put explicitly in the regressions the name of the variable followed by “normed”.**

4. I am unhappy with various minor aspects of this revision: The authors’ claim to defend their sample size of 50 participants “in studies with similar sample sizes (1,2,4,8)” (p. 6) is misleading readers because only Experiment 2 of ref. 4 has 55 participants, while all others have between 16 and 27.

**We dropped the expression “similar sample size” to the actual range of 16 to 55. Thus, our sample size is on the larger end.**

My request to determine power or sensitivity retrospectively was not addressed.

**We calculated power, using response times from our participants as it is known in the literature that confidence and response times correlate (Participants: Page 7). Overall, the obtained power is large.**

Some wording is still poor: On page 7 “Exp. 1 was run in a 13-inch laptop” (should be “on”),

**Solved.**

or “Uncertainty is a more general concept as it is not a conditioned in choice.” (p. 5) is ungrammatical.

**We dropped that expression and rather clarify the distinction of uncertainty and confidence in the model because it is more concrete for the reader: DDM model: Page 13: “It is important to highlight that uncertainty UN is not the same as confidence in the model. UN is the base standard deviation of the inference, while confidence is the cumulative probability that μdv is greater than zero after a choice is made (i.e. after dv arrives at one of the thresholds).”**

Several references contain the superfluous word “internet”.

**We did not realize that Mendeley was doing that, sorry.**

OTHER IMPORTANT CLARIFICATIONS

- **In Exp. 3, by mistake, we standardized to a value that was not the peak pressure in general but the initial peak value in the Arduino signal greater than a low threshold. We corrected this oversight and that is why some regression estimates of Exp. 3 are different in this current revision. Importantly the overall pattern/direction/significance of the effects is identical, just slightly different estimates (i.e. both constants are correlated because each trial is a time series). This oversight did not affect the conclusions and the corrections, if any, consolidated the overall results.**

**The oversight happened because in Exp. 3 we decided to change the serial function used by Matlab to communicate with the Arduino (it will be deprecated; the new one is serialport). When we changed the code we marked events slightly differently. During data analyses we missed changing some of the marks and we ended up using, mistakenly, the peak pressure across trials of the first pressure signal sent by the Arduino greater than a low-pressure threshold on each trial. As we mentioned, we corrected this in the current revision.**

- **In the Table 1 of the previous revision we didn’t include the variable controlling for error feedback. In this revision we fixed that.**
- **The only tables that include interaction effects are the ones theoretically relevant. In Page 11: “We included interactions in the regressions when it was theoretically relevant. Namely when the dependent variable was response times (distinct slopes by numeral type suggest different mental magnitudes) and pressure (Figure 1, central panel shows distinct slopes for correct and incorrect trials).”**

Reviewer #2:

The authors have addressed most of the raised points. In my previous revision I focused on the model, as it is interesting in my opinion and authors have included a wider reflection on this issue
