# Supplementary material for: Reduced choice-confidence in negative numerals
Source: PLoS One. 2022 Oct 3;17(10):e0272796. doi: 10.1371/journal.pone.0272796 (PMC9529092; doi:10.1371/journal.pone.0272796)
Supplement: Attachment — Submitted filename: Reviewers_3_PONE.docx [file pone.0272796.s005.docx]

PONE-D-21-25521R3

Reduced choice-confidence in negative numerals

PLOS ONE

Dear Dr. Alonso-Diaz,

Thank you for submitting your manuscript to PLOS ONE. After careful consideration, we feel that it has merit but does not fully meet PLOS ONE’s publication criteria as it currently stands. Therefore, we invite you to submit a revised version of the manuscript that addresses the points raised during the review process.

One of the original reviewers (#1) suggested rejection, based on unsatisfactorily response to his/her comments. I involved a third reviewer. While generally praising your work, reviewer #3 raised a number of further concerns.

I encourage the authors to address all the new criticism raised by reviewer #3, as well as trying to address the  residual part of comments of #1. Please include a response to reviewer covering also #1.

Please submit your revised manuscript by Aug 13 2022 11:59PM. If you will need more time than this to complete your revisions, please reply to this message or contact the journal office at [plosone@plos.org](mailto:plosone@plos.org). When you're ready to submit your revision, log on to https://www.editorialmanager.com/pone/ and select the 'Submissions Needing Revision' folder to locate your manuscript file.

Please include the following items when submitting your revised manuscript:

- A rebuttal letter that responds to each point raised by the academic editor and reviewer(s). You should upload this letter as a separate file labeled 'Response to Reviewers'.
- A marked-up copy of your manuscript that highlights changes made to the original version. You should upload this as a separate file labeled 'Revised Manuscript with Track Changes'.
- An unmarked version of your revised paper without tracked changes. You should upload this as a separate file labeled 'Manuscript'.

If you would like to make changes to your financial disclosure, please include your updated statement in your cover letter. Guidelines for resubmitting your figure files are available below the reviewer comments at the end of this letter.

If applicable, we recommend that you deposit your laboratory protocols in protocols.io to enhance the reproducibility of your results. Protocols.io assigns your protocol its own identifier (DOI) so that it can be cited independently in the future. For instructions see: [https://journals.plos.org/plosone/s/submission-guidelines#loc-laboratory-protocols](https://nam10.safelinks.protection.outlook.com/?url=https%3A%2F%2Fjournals.plos.org%2Fplosone%2Fs%2Fsubmission-guidelines%23loc-laboratory-protocols&data=05%7C01%7Calonsosantiago%40javeriana.edu.co%7C80602985f1e64cea950108da59f5d4b4%7Cdaf7990e8a3f409c9b762a5475098000%7C0%7C0%7C637921209640555429%7CUnknown%7CTWFpbGZsb3d8eyJWIjoiMC4wLjAwMDAiLCJQIjoiV2luMzIiLCJBTiI6Ik1haWwiLCJXVCI6Mn0%3D%7C3000%7C%7C%7C&sdata=r0tZZTe20p9UzTU55ZpjeMiUC9MWiQUjxN8qhoiVwfI%3D&reserved=0). Additionally, PLOS ONE offers an option for publishing peer-reviewed Lab Protocol articles, which describe protocols hosted on protocols.io. Read more information on sharing protocols at [https://plos.org/protocols?utm_medium=editorial-email&utm_source=authorletters&utm_campaign=protocols](https://nam10.safelinks.protection.outlook.com/?url=https%3A%2F%2Fplos.org%2Fprotocols%3Futm_medium%3Deditorial-email%26utm_source%3Dauthorletters%26utm_campaign%3Dprotocols&data=05%7C01%7Calonsosantiago%40javeriana.edu.co%7C80602985f1e64cea950108da59f5d4b4%7Cdaf7990e8a3f409c9b762a5475098000%7C0%7C0%7C637921209640555429%7CUnknown%7CTWFpbGZsb3d8eyJWIjoiMC4wLjAwMDAiLCJQIjoiV2luMzIiLCJBTiI6Ik1haWwiLCJXVCI6Mn0%3D%7C3000%7C%7C%7C&sdata=5LbKcVM0hteo8PZ1Kd4nnw84phDbed2c9n%2BuPlsGdgk%3D&reserved=0).

We look forward to receiving your revised manuscript.

Kind regards,

Federico Giove, PhD
Academic Editor
PLOS ONE

[Note: HTML markup is below. Please do not edit.]

Reviewers' comments:

Reviewer's Responses to Questions

**Comments to the Author**

1. If the authors have adequately addressed your comments raised in a previous round of review and you feel that this manuscript is now acceptable for publication, you may indicate that here to bypass the “Comments to the Author” section, enter your conflict of interest statement in the “Confidential to Editor” section, and submit your "Accept" recommendation.

Reviewer #3: (No Response)

2. Is the manuscript technically sound, and do the data support the conclusions?

The manuscript must describe a technically sound piece of scientific research with data that supports the conclusions. Experiments must have been conducted rigorously, with appropriate controls, replication, and sample sizes. The conclusions must be drawn appropriately based on the data presented.

Reviewer #3: Yes

3. Has the statistical analysis been performed appropriately and rigorously?

Reviewer #3: Yes

4. Have the authors made all data underlying the findings in their manuscript fully available?

The [PLOS Data policy](https://nam10.safelinks.protection.outlook.com/?url=http%3A%2F%2Fwww.plosone.org%2Fstatic%2Fpolicies.action%23sharing&data=05%7C01%7Calonsosantiago%40javeriana.edu.co%7C80602985f1e64cea950108da59f5d4b4%7Cdaf7990e8a3f409c9b762a5475098000%7C0%7C0%7C637921209640555429%7CUnknown%7CTWFpbGZsb3d8eyJWIjoiMC4wLjAwMDAiLCJQIjoiV2luMzIiLCJBTiI6Ik1haWwiLCJXVCI6Mn0%3D%7C3000%7C%7C%7C&sdata=U4Mq%2FxvGA1N56kr0x4AdZo4UDGagc2EgQr4NSqbbVIs%3D&reserved=0) requires authors to make all data underlying the findings described in their manuscript fully available without restriction, with rare exception (please refer to the Data Availability Statement in the manuscript PDF file). The data should be provided as part of the manuscript or its supporting information, or deposited to a public repository. For example, in addition to summary statistics, the data points behind means, medians and variance measures should be available. If there are restrictions on publicly sharing data—e.g. participant privacy or use of data from a third party—those must be specified.

Reviewer #3: Yes

5. Is the manuscript presented in an intelligible fashion and written in standard English?

PLOS ONE does not copyedit accepted manuscripts, so the language in submitted articles must be clear, correct, and unambiguous. Any typographical or grammatical errors should be corrected at revision, so please note any specific errors here.

Reviewer #3: Yes

6. Review Comments to the Author

#REVIEWER 3

Please use the space provided to explain your answers to the questions above. You may also include additional comments for the author, including concerns about dual publication, research ethics, or publication ethics. (Please upload your review as an attachment if it exceeds 20,000 characters)

Reviewer #3: This manuscript deals with a question that, in my opinion, looks highly relevant for many applications: do negative numbers induce more uncertainty than positive ones? It does so by resorting to different strategies: hardware that allows graded responses in the form of pressure, cognitive modelling with the diffusion model… In general, I like the idea behind this research. However, I had problems to fully understand what the manuscript is describing, in part because the text organization is not clear.

**Response:** **Thanks for your positive words, we appreciate them. We hope that we address your concerns in this revised manuscript.**

I will describe next a few comments that may help to improve the manuscript:
1. Text organization
I found the Introduction well written and easy to follow. Not being an expert myself, I think that the Introduction contains the basic information needed to understand the purpose and justification of the studies.
The rest of the manuscript is organized in a way that, in my opinion, does not help to understand what is being described:
-The three experiments are described at the same time, which makes it difficult to understand the differences between them and why one needs to conduct three experiments at all. A more traditional organization with each experiment being described (together with its justification) alone would be better, in my opinion. If the experiments have very similar procedure, you could just refer to that explanation in previous sections. Now the motivation to run Experiment 3, for instance, seems completely overlooked (it is mentioned somewhere at the beginning and it seems to never matter again).

**Response: In the new manuscript we separated each experiment into different sections. Now the motivation for each one is explained clearly at the beginning of each one. Thus, Exp. 1 is traditional keyboard experiment that allow us to show some confidence predictions from the drift-diffusion model (DDM). Exp. 2 and 3 confirm one of those predictions and replicates the DDM results. Exp. 3 further controls for important methodological aspects.**

-The same happens with the Data Analysis, Drift-Diffusion model, and Results sections. It is hard to keep in mind all the information when one goes through these sections. The Results section, for instance, is just a collection of tables, full of numbers, with little guidance in the text. I would prefer a more traditional organization: experiment-wise, with a description of the data analyses that will be later reported (and explained!) in the Results section.

**Response: Now each experiment has its own data analysis and results sections. The drift diffusion is only explained in Exp. 1. By separating each experiment we think that now the explanations are clearer.**

Maybe, too, a final section for the modelling that could include the three experiments, if you want to. Or perhaps the diffusion model can be described in the first Data Analysis section, and then in subsequent subsections of the Results sections for each experiment. Now it is all a bit too mixed up.

**Response: We now describe the drift diffusion model in the first Data Analysis (in Exp. 1) and then presented the results for each experiment on their own subsection.**

2. Theoretical implications.
Admittedly, I am not an expert on numerical cognition. From my reading of the Introduction, I got that there are two competing theories that make different predictions concerning negative numbers. But it is not clear to me whether and how the current studies help in addressing these questions. Would it be possible to connect the results to these theories, perhaps favouring one over the other? (It seems that the diffusion model results indicate that differences between numbers are not an encoding effect)

**Response: The results indeed point to holistic representations of numbers, not merely encoding. The diffusion model, and the response times patterns in Exp. 1 and 2, surely suggests that. In the discussion section we are now more explicit on saying this (Page 32.)**

My impression is that, despite the highly interesting theoretical debate, the results of these experiments are just descriptive: they suggest that there is an uncertainty burden in negative numbers, at least in this task. Thus, we cannot advance too much in theory without further research.

**Response: Indeed, button pressure alone cannot disentangle which theory is correct (at least with our design). We mention this in Page 32. Still response times and the drift diffusion model favor holistic representations and we mention this also in Page 32. However, given the assumptions behind the drift-diffusion model we are still conservative and mention that further research is required.**

I was curious about a potential extension of these results to different tasks. Would negative numbers produce more uncertain answers in any type of task? For instance: Here, the task is very simple and implies recognizing negative numbers. I don’t know if the result is generalizable to production tasks. What would happen if, facing any numerical task (solving mathematical problems, or just emitting judgments), those responses that are negative produce more uncertainty as well?

**Response: in this new revision the last phrase is suggesting such critical future questions.**

3. Minor comments:
-Power analyses. The authors conduct post hoc power analyses. This is not recommended (Althouse, 2021; Hoenig & Heisy, 2001). Post hoc power estimations are *determined* by the p-value. So, once you get a significant result, what is the point in computing power?
What can we do, then? Ideally, power calculations should be conducted *before* data collection (“a priori power analyses”), so that you determine you sample size given an estimated effect size and a desired power level. However, this is not easy to do as the effect size must still be estimated (which is hard to do, as the literature is often biased).
Thus, I assume that you did not conduct power analyses a priori. Then, what you can do is conducting sensitivity analyses. Sensitivity analyses can be conducted a priori or a posteriori, and they reach a compromise between power goals and practical issues. You can use G*Power or any other software to: (1) decide which sample size you are willing to collect (or you have already collected) for practical/ethical/economic reasons, (2) fix a power level (e.g., 80%), and (3) solve for the minimal effect size that you can detect.
If the sensitivity analyses reveal that you can detect reliably (with good power) even small effects, then your study is well powered for those effects. If you can only detect large effects, that means that you have low power for small effects.
Another thing you can do is reporting effect sizes and confidence intervals. Even if the results are significant, a large interval suggests that the study is not informative.

Response: We fully agree with you. In the original manuscript we did not do post-hoc justifications. As the review process advanced, we were trying to understand the requirements of previous reviewers. We agree with you that the sensitivity analysis is a good middle ground. We now provide a sensitivity analysis for Exp. 1. For Exp. 2 we mention that we preregistered a max. sample size and that Exp. 3 also follow that cap.

-Lots of t-tests were conducted (e.g. with diffusion model parameters, Table 5) without (as far as I can see) multiple contrasts protection.

**Response: We corrected for multiple comparisons with Holm-Sidak (page 12)**

- P. 18: “However, the term…” (do you mean “interaction term”?)

**Response: Yes, you are right, we corrected that.**

References:
Althouse A. D. (2021). Post Hoc Power: Not Empowering, Just Misleading. The Journal of surgical research, 259, A3–A6. https://doi.org/10.1016/j.jss.2019.10.049
Hoenig JM, Heisey DM. The abuse of power: the pervasive fallacy of power calculations for data analysis. Am Stat. 2001; 55:19-24.

7. PLOS authors have the option to publish the peer review history of their article ([what does this mean?](https://nam10.safelinks.protection.outlook.com/?url=https%3A%2F%2Fjournals.plos.org%2Fplosone%2Fs%2Feditorial-and-peer-review-process%23loc-peer-review-history&data=05%7C01%7Calonsosantiago%40javeriana.edu.co%7C80602985f1e64cea950108da59f5d4b4%7Cdaf7990e8a3f409c9b762a5475098000%7C0%7C0%7C637921209640555429%7CUnknown%7CTWFpbGZsb3d8eyJWIjoiMC4wLjAwMDAiLCJQIjoiV2luMzIiLCJBTiI6Ik1haWwiLCJXVCI6Mn0%3D%7C3000%7C%7C%7C&sdata=%2FVQYXFvs6%2BS2gZYrdDCRbb71ImuhcNHSOXDoyNW%2B2JY%3D&reserved=0)). If published, this will include your full peer review and any attached files.


If you choose “no”, your identity will remain anonymous but your review may still be made public.


**Do you want your identity to be public for this peer review?** For information about this choice, including consent withdrawal, please see our [Privacy Policy](https://nam10.safelinks.protection.outlook.com/?url=https%3A%2F%2Fwww.plos.org%2Fprivacy-policy&data=05%7C01%7Calonsosantiago%40javeriana.edu.co%7C80602985f1e64cea950108da59f5d4b4%7Cdaf7990e8a3f409c9b762a5475098000%7C0%7C0%7C637921209640555429%7CUnknown%7CTWFpbGZsb3d8eyJWIjoiMC4wLjAwMDAiLCJQIjoiV2luMzIiLCJBTiI6Ik1haWwiLCJXVCI6Mn0%3D%7C3000%7C%7C%7C&sdata=%2FSFSiVITB7E8%2BsA39UE4gcQWJzCA9z1WmRieQTZyVIc%3D&reserved=0).

Reviewer #3: No

Reviewer #1:

**NOTE: THIS IS FROM THE PREVIOUS ROUND (WITH MINOR EDITS). WE INCLUDE THE BY THE EDITOR’S REQUEST.**

1. The authors made several adjustments and additions that strengthen the ms, including explanations of their idiosyncratic terminology (“dummy trials” for filler trials) and references to relevant papers that were previously omitted. However, repeatedly directing readers to Ganor-Stern & Tzelgov (2008) for the component model is still suboptimal because the paper by Huber (their Ref 5) constitutes its more recent development.

**We now extend the component model explanation with the Huber et al neural network (Intro.: Pages 3-4).**

Similarly, the introduction of “button pressure” as the measure of interest (on page 4) is immediately followed by references to “motor metrics”, i.e. kinematic studies, even though the “button pressure” is an isometric assessment without any kinematics involved. In my idiosyncratic view these are suboptimal revisions.

**We wanted to show to a general audience that “motor metrics” are used in cognition. We understand that those references were about kinematics. Thus, we now drop those kinematics references to avoid any confusion.**

2. More importantly, the methods descriptions are still not detailed enough to permit replication. Although stimulus ranges are now clear and the recording apparatus for “button pressure” is now explained in more detail, the sensitivity of this device is still unclear. If the range is 0.25 to 0.90, does this mean that 65 levels of pressure were discriminable

**The range 0.25-0.90 is continuous. There are more than 65 discrete levels.**

**The actual range from the raw data is 0.025 to 1 (Exp 2) and 0.05 to 1 (Exp. 3). The 0.25 – 0.9 range was after taking averages per numerical distances. In the new manuscript (Data analysis) we decided that it was clearer if we present the ranges from the raw data because the right end is 1 and the left range is close to zero.**

while the actual force produced (in Pascal or Newton) remains unknown?

**The sensor detects pressure as a change in resistance. The details of the resistors and Arduino wiring are in the manuscript and supplemental information. The Arduino transforms those changes of resistance into a discrete signal between 0 and 1023. All subjects used the same Arduino setup (including voltage and resistors). Thus, we do not report Pascals or Newtons (the transformation from change in resistance to Newtons or Pascals is not trivial). Still, the change in resistance indeed relates to force; resistance-based pressure is used in many current devices including mobile phones and many portable devices.**

Another open issue, of fundamental importance for the later discussion about early attentional vs later conceptual effects: What was the sampling frequency (i.e. the temporal resolution of pressure measurements)?

**We now report the sampling frequency of the Arduino board in the manuscript (Apparatus, page 7-8) (approx. 26 hz but see manuscript for details).**

**It is not fast enough to fully disentangle early attention and later concepts (holistic magnitudes).**

**In the discussion we accept that it could be attention, but it would require some ad-hoc accommodations so that all our tables and results are about attention and not confidence.**

**we do think that confidence results speak about concepts, namely the existence of negative holistic magnitudes (see discussion section). However, we are also aware that our paper only brings partial clarity to negative magnitude processing. Our paper is just about confidence.**

3. Moreover, despite my extensive queries on this point, I still find only a SINGLE sentence that describes the force data analysis, namely “Data analysis. We used panel linear regressions to analyze the accuracy (linear probability model), response times up to two standard deviations from the mean (i.e., 95% of the trials), and button pressure”.

**We apologize for our lack of clarity; it is not our intention. In the first revision we tried to address all your comments, but we clearly fail. We hope that our responses below clarify our data analysis.**

This leaves open fundamental questions such as the time during which force was recorded or integrated,

**We analyzed signals as soon as the numerals appeared on screen and until the subject selected an option and was no longer pressing the force sensor (i.e. force resistance < low_pressure_threshold) (this for all trials). (Data Analysis).**

the data filtering or trimming for this inherently noisy signal,

**We did not filter Arduino signals. When the sensor is pressed it sends a reading to Matlab based on the participant’s pressure. In the figure below (see the .docx document rather than the Editorial Manager pdf), each trial has a stereotypical look: a peak. This means that participants had a brief contact with the sensor** **(here we plot the raw Arduino signal, before any standardization) (see all raw data in Supplemental Figures 18 and 19).**

**We filtered data on the behavior side. We reported in the manuscript two criteria: response time greater than 2 standard deviations from the mean of all the data and accuracy less than 85%.**

**We did detect an anomaly thanks to your comment. We plotted all the raw pressure data and we detected one subject who used the keyboard instead of the Arduino sensors. The pressures of subj. 33443 (Exp. 2) were always at baseline levels (Supp. Figure 18). This subj did respond accurately to the task, meaning that they used the keyboard (we explain this on the participants section of the new manuscript).**

**The reason this could happen was that we coded the first experiment (Exp. 1) so that subjects had to use the keyboard. In Exp. 2 we used the same base code (plus the Arduino stuff) and even though we verbally explained to all subjects to use the Arduino sensors, we kept, by mistake, the original instructions of Exp. 1 on screen. Subj. 33443 was the only subject (across Exp. 2 and 3) who mistakenly used the keyboard (see Supplemental Figure 18). We excluded this subject from this revision. Importantly, the main pattern of results did not change.**

or the computation of parameters for analysis, such as average or peak force per trial, or many other candidates. None of this is explained.

**For this revision we now provide a concrete example for further clarity (Data Analysis section). Specifically, we used for analysis peak force during the trial, normalized to the max peak force during the whole session as a normalizing constant (separately for the left and right sensor). For instance, if subject X max peak force to the left during his whole session was 984, then all left pressures during the session were divided by 984. We used this metric in the tables. That is, the regression parameters were obtained with standardized peak pressure. We now put explicitly in the regressions the name of the variable followed by “normed”.**

4. I am unhappy with various minor aspects of this revision: The authors’ claim to defend their sample size of 50 participants “in studies with similar sample sizes (1,2,4,8)” (p. 6) is misleading readers because only Experiment 2 of ref. 4 has 55 participants, while all others have between 16 and 27.

**We dropped the expression “similar sample size” to the actual range of 16 to 55. Thus, our sample size is on the larger end.**

My request to determine power or sensitivity retrospectively was not addressed.

**We calculated power, using response times from our participants as it is known in the literature that confidence and response times correlate (Participants: Page 7). Overall, the obtained power is large.**

Some wording is still poor: On page 7 “Exp. 1 was run in a 13-inch laptop” (should be “on”),

**Solved.**

or “Uncertainty is a more general concept as it is not a conditioned in choice.” (p. 5) is ungrammatical.

**We dropped that expression and rather clarify the distinction of uncertainty and confidence in the model because it is more concrete for the reader: DDM model: Page 13: “It is important to highlight that uncertainty UN is not the same as confidence in the model. UN is the base standard deviation of the inference, while confidence is the cumulative probability that μdv is greater than zero after a choice is made (i.e. after dv arrives at one of the thresholds).”**

Several references contain the superfluous word “internet”.

**We did not realize that Mendeley was doing that, sorry.**

OTHER IMPORTANT CLARIFICATIONS

- **In Exp. 3, by mistake, we standardized to a value that was not the peak pressure in general but the initial peak value in the Arduino signal greater than a low threshold. We corrected this oversight and that is why some regression estimates of Exp. 3 are different in this current revision. Importantly the overall pattern/direction/significance of the effects is identical, just slightly different estimates (i.e. both constants are correlated because each trial is a time series). This oversight did not affect the conclusions.**

**The oversight happened because in Exp. 3 we decided to change the serial function used by Matlab to communicate with the Arduino (it will be deprecated; the new one is serialport). Exp. 3 was the experiment that reviewer 1 asked i.e. it was a new experiment and when we changed the code to include the new conditions and Matlab function, the code we wrote marked events slightly differently. During data analyses we missed changing some of the marks and we ended up using, mistakenly, the peak pressure across trials of the first pressure signal sent by the Arduino greater than a low-pressure threshold on each trial. As we mentioned, we corrected this in the current revision.**

- **In the Table 1 of the previous revision we didn’t include the variable controlling for error feedback. In this revision we fixed that.**
- **The only tables that include interaction effects are the ones theoretically relevant. In Page 11: “We included interactions in the regressions when it was theoretically relevant. Namely when the dependent variable was response times (distinct slopes by numeral type suggest different mental magnitudes) and pressure (Figure 1, central panel shows distinct slopes for correct and incorrect trials).”**
- **The manuscript is on psyarxiv with figures on the manuscript (https://psyarxiv.com/sdfb9/)**
